# Solvothermal synthesis of cobalt PCP pincer complexes from [Co_2_(CO)_8_]

**DOI:** 10.1007/s00706-023-03123-x

**Published:** 2023-09-09

**Authors:** Heiko Schratzberger, Daniel Himmelbauer, Wolfgang Eder, Michael Weiser, Berthold Stöger, Karl Kirchner

**Affiliations:** 1 Institute of Applied Synthetic Chemistry, TU Wien, Getreidemarkt 9/163-AC, 1060 Vienna, Austria; 2 X-Ray Center, TU Vienna, Getreidemarkt 9/163-AC, 1060 Wien, Austria

**Keywords:** Pincer complexes, Cobalt, Transmetalation, Oxidative addition

## Abstract

Treatment of [Co_2_(CO)_8_] with the *ipso*-substituted P(C-X)P^Y^ ligands (X = Br, Cl; R = *i*Pr, *t*Bu) bearing Y = NH and CH_2_ linkers under solvothermal conditions affords the five-coordinate Co(I) and Co(III) complexes [Co^I^(PCP^Y^-R)(CO)_2_] and [Co^III^(PCP^Y^-R)X_2_]. The later are paramagnetic exhibiting a solution magnetic moment in the range of 3.0–3.3 μ_B_ which is consistent with a d^6^ intermediate spin system corresponding to two unpaired electrons. In the case of P(C-X)P^Y^ ligands (X = Br, Cl; R = *t*Bu; Y = NH) the formation of the square planar Co(II) complex [Co(PCP^NH^-*t*Bu)X] was favored. This complex gives rise to a magnetic moment of 1.8 μ_B_ being consistent with a d^7^ low spin system corresponding to one unpaired electron. All complexes are characterized by means of spectroscopic techniques (NMR, IR), HR-MS. Representative complexes were also characterized by X-ray crystallography.

**Graphical abstract:**

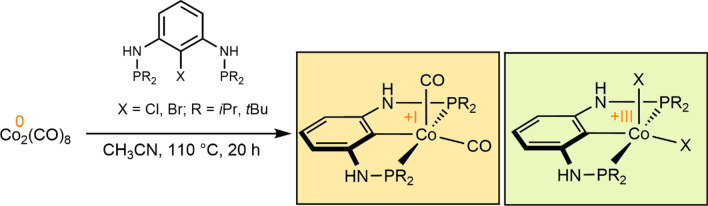

**Supplementary Information:**

The online version contains supplementary material available at 10.1007/s00706-023-03123-x.

## Introduction

PCP pincer complexes which often feature an aromatic anionic benzene backbone connected to phosphine donors via CH_2_, O, or NR (R = H, alkyl, aryl) linkers and a metal–carbon single bond are a very important class of compounds [1–17]. Modifications of the substituents at the donor sites and/or the spacers enables the modification of electronic, steric and even stereochemical parameters which allows often the generation of highly active catalysts for a range of chemical transformations with high selectivity.

With respect to cobalt, PCP complexes are comparatively rare. This may be attributed to the failure of many simple metal salts to cleave the C-H bonds of the arene moiety of the pincer ligands and/or the thermodynamic instability of the resulting complexes.

Common motifs of cobalt PC*sp*^*2*^P and PC*sp*^*3*^P pincer complexes, which serve as synthetic entries into Co PCP pincer chemistry are depicted in Scheme 1. These are typically derived from simple Co(0), Co(I), and Co(II) precursors such as [Co(PMe_3_)_4_], [Co_2_(CO)_8_], [CoCl(PMe_3_)_3_], [Co(Me)-(PMe_3_)_4_], [Co(N(SiMe_3_)_2_)_2_(py)_2_], and anhydrous CoX_2_ (X = Cl, Br, I).

**Figure:**
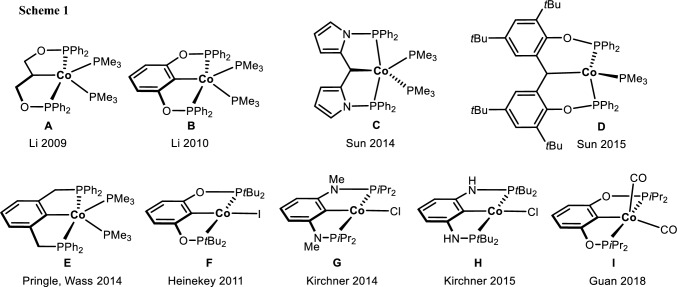

The first cobalt PCP pincer complexes were synthesized by Li and co-workers in 2009 [18, 19]. This was achieved by the activation of sp^3^ and sp^2^ C-H bonds initiated by the electron-rich cobalt complex [Co(Me)(PMe_3_)_4_] in reaction with (Ph_2_POCH_2_)_2_CH_2_ and 2,6-(Ph_2_PO)_2_C_6_H_4_, respectively, which led to the formation of the Co(I) PCP complexes **A** and **B** (Scheme 1). This process is accompanied by liberation of methane and PMe_3_ which is the driving force of this reaction. The successful use of [Co(Me)(PMe_3_)_4_] to activate sp^3^ C–H and sp^2^ C–H bonds was also applied to other pincer systems. For instance, Sun and co-workers described the coordination chemistry of cobalt complexes **C** and **D** with a new PC*sp*^*3*^P pincer ligand based on a dipyrrolmethane backbone [20, 21]. Pringle and co-workers reported the synthesis of the Co(I) PC*sp*^*2*^P complex **E** by a transmetalation reaction between 1-lithio-2,6-bis[(diphenylphosphino)-methyl]benzene and [Co(Cl)(PMe_3_)_3_] [22]. The Co(II) iodide complex **F** bearing a benzene-centered P^O^C^O^P pincer ligand was reported by Heinekey [23, 24]. Kirchner and co-workers [25, 26] reported on the synthesis and reactivity of a series of Co(I), Co(II), and Co(III) PCP complexes bearing pincer ligands based on the 1,3-diaminobenzene scaffold. Treatment of anhydrous CoCl_2_ with the PCP ligand in the presence of *n*-BuLi in THF affords the 15e^−^ complexes [Co(PC*sp*^*2*^P^Me^-*i*Pr)Cl] (**G)** and [Co(PC*sp*^*2*^P^Me^-*t*Bu)Cl] (**H**) (Scheme 1). Guan and coworkers adapted the oxidative addition approach and used the Co(0) complex [Co_2_(CO)_8_] as precursor for the oxidative addition of 2,6-(Ph_2_PO)_2_C_6_H_4_ to afford complexes of the type **I** [27].

Herein, we report on the synthesis and characterization of a series of cobalt PCP pincer complexes in the formal oxidation states + I, + II, and + III. The new PCP cobalt complexes were obtained by treatment of the Co(0) precursor [Co_2_(CO)_8_] with *ipso*-substituted P(C_ipso_-X)P^Y^ ligands (X = Br, Cl; R = *i*Pr, *t*Bu) bearing Y = NH and CH_2_ linkers under solvothermal conditions.

## Results and discussion

When a suspension of [Co_2_(CO)_8_] with P(C-X)P^NH^-*i*Pr (X = Cl (**1a**) or Br (**1b**)) in acetonitrile was placed into a sealed microwave glass vial and stirred for 20 h at 110 °C the Co(I) and Co(III) complexes [Co(PCP^NH^-*i*Pr)(CO)_2_] (**4**) and [Co(PCP^NH^-*i*Pr)Cl_2_] (**6a**) or [Co(PCP^NH^-*i*Pr)Br_2_] (**6b**), respectively, were formed in an approximately 1:1 ratio (Scheme 2). Shorter reaction times did not alter the outcome of this reaction but resulted in lower conversions. After workup, complexes **4** and **6a** or **6b** were obtained in 43, 42, and 46%, respectively. The reaction with the analogous *t*Bu ligands P(C-X)P^NH^-*t*Bu (**1c**, **1d**) led to the formation of complexes [Co(PCP^NH^-*t*Bu)(CO)_2_] (**5**) and [Co(PCP^NH^-*t*Bu)-Cl_2_] (**6c**) or [Co(PCP^NH^-*t*Bu)Br_2_] (**6d**) (Scheme 2). However, in contrast to complex **4**, complex **5** could not be isolated in pure form due to rapid loss of CO accompanied by the additional formation of intractable paramagnetic materials. Complex **4** was characterized by means of NMR spectroscopy, IR spectroscopy, and HR-MS.

**Figure:**
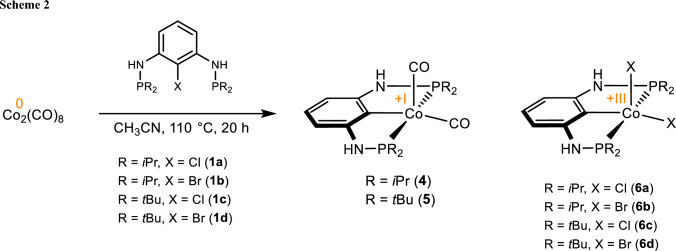

Complex **4** displays two strong absorption bands in the IR spectrum observed at 1896 and 1956 cm^−1^, respectively, for the mutually *cis* CO ligands assignable to the symmetric and asymmetric CO stretching frequencies. In the ^13^C{^1^H} NMR spectrum the CO ligand gives rise to a broad low-field resonance at 207.4 ppm. The *ipso*-carbon exhibits a triplet at 133.1 ppm (*J*_*PC*_ = 17.9 Hz). The transient formation of **5** was detected by IR spectroscopy exhibiting two strong absorption bands 1903 and 1958 cm^−1^, respectively.

**Fig. 1:**
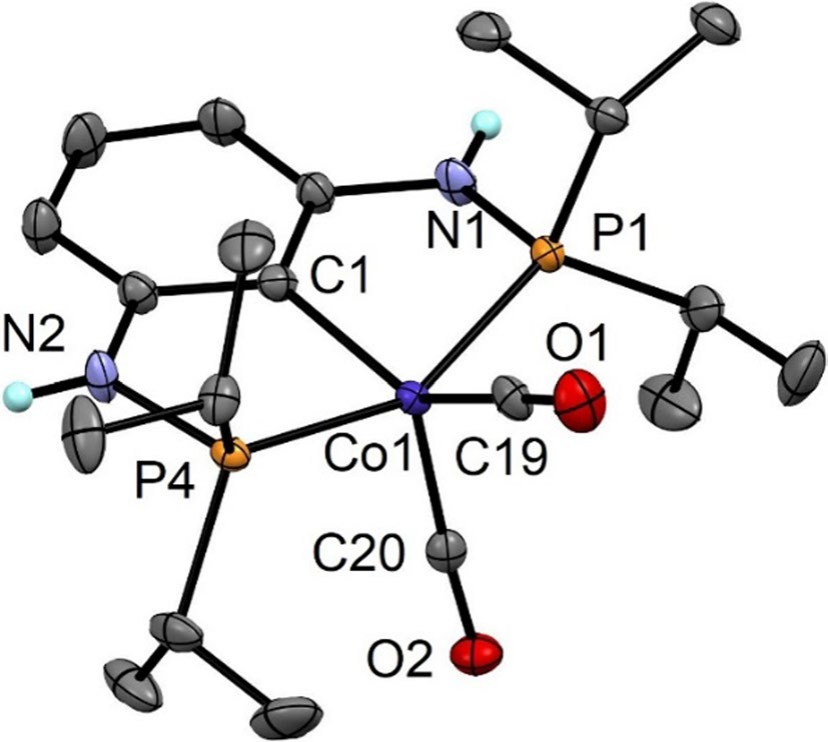
Structural view of [Co(PCP^NH^-*i*Pr)(CO)_2_] (**4**) showing 50% thermal ellipsoids (most H atoms omitted for clarity). Selected bond lengths (Å) and bond angles (deg): Co1-C1 2.022(3), Co1-P1 2.1807(7), Co1-P4 2.1827(6), Co1-C19 1.752(3), Co1-C20 1.782(2), P1-Co1-P4 153.46(3), C19-Co1-C20 108.4(1), C1-Co1-C19 141.1(1), C1-Co1-C20 110.5(1)

The solid-state structure of **4** was established by single-crystal X-ray diffraction. A molecular view is depicted in Fig. 1 with selected bond distances given in the captions. This complex adopts basically a distorted square-pyramidal geometry with C19-Co1-C20 and C1-Co1-C19 angles of 108.4(1) and 141.1(1)°, respectively. The structural parameter τ_5_ is 0.206 (τ_5_ = 0 indicates an ideal square pyramidal structure) [28]. The P1-Co1-P1 angle is 153.46(2)°. The CO ligands do not deviate significantly from linearity with Co1-C19-O1 and Co1-C20-O2 angles of 178.1(2) and 171.8(2)°, respectively. The structure of **4** is very similar to [Co(PCP^NMe^-*i*Pr)(CO)_2_] bearing NMe linkers [26], but differs from the structure of the related complex [Co(PCP^CH2^-Ph)(CO)_2_] which adopts a distorted trigonal bipyramidal geometry with an unusually small P-Co-P angle of 134.6(1)° [22].

**Fig. 2:**
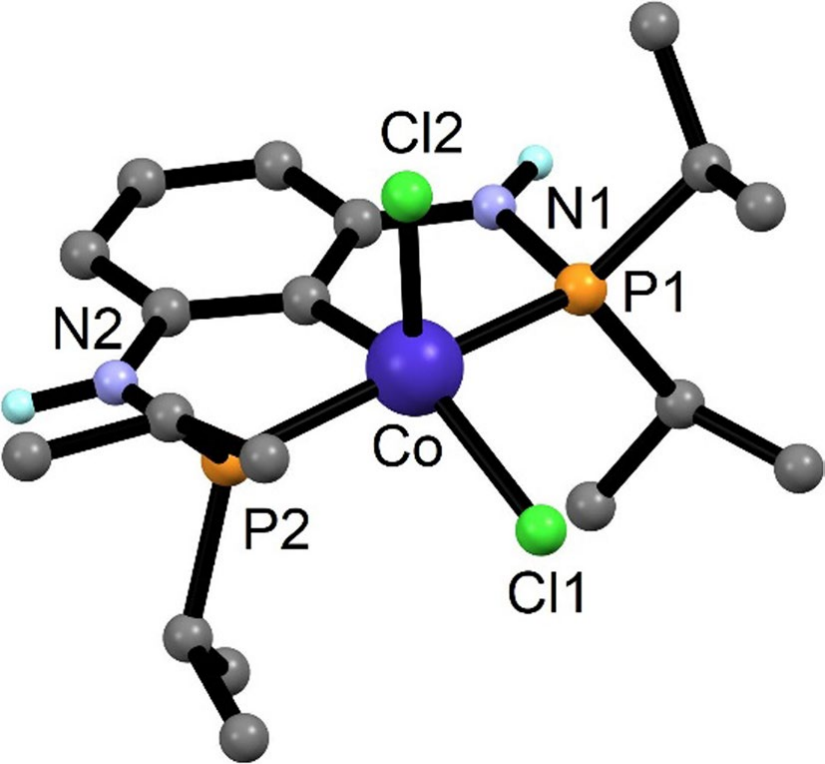
DFT calculated structure of [Co(PCP^NH^-*i*Pr)(Cl)_2_] (**6a**) in the triplet state (S = 1). Selected bond lengths (Å) and bond angles (deg): Co-C1 1.911, Co-P1 2.258, Co-P2 2.2251, Co-Cl1 2.273, Co-Cl2 2.294, P1-Co-P2 164.7, Cl1-Co-Cl2 108.7

Complexes **6a**–**6d** are paramagnetic. Measurements of the magnetic susceptibility in solution (Evans method [29], benzene) gave *µ*_eff_ values of 3.1(8), 3.2(3), 3.2(9), and 3.3(5) µ_B_, respectively, which corresponds to two unpaired electrons and a formal oxidation state of + III. These values are within the observed range of other five-coordinate Co(III) complexes known [25, 30]. In agreement with experiment, DFT calculations reveal that the triplet state (S = 1) of **6a** (depicted in Fig. 2) is more stable than corresponding low-spin state with S = 0 by 33.1 kJ mol^−1^. Complex **6a** shows the metal in a distorted-square pyramidal conformation which is typical for five-coordinate Co(III) complexes in this spin state [25, 30].

Performing the reaction with P(C-X)P^NH^-*t*Bu at 130 °C instead of 110 °C resulted in the formation of the square planar Co(II) complexes [Co(PCP^NH^-*t*Bu)Cl] (**7c**) or [Co(PCP^NH^-*t*Bu)Br] (**7d**). These compounds were isolated in 89 and 91% yields (Scheme 3). The solution magnetic moments of 1.8(2) and 1.8(3) μ_B_ (benzene, Evans method) are consistent with a d^7^ low spin system corresponding to one unpaired electron. It has to be noted that **7c** was already prepared recently by reacting anhydrous CoCl_2_ with PCP^NH^-*t*Bu in the presence of *n*-BuLi [26].

**Figure:**
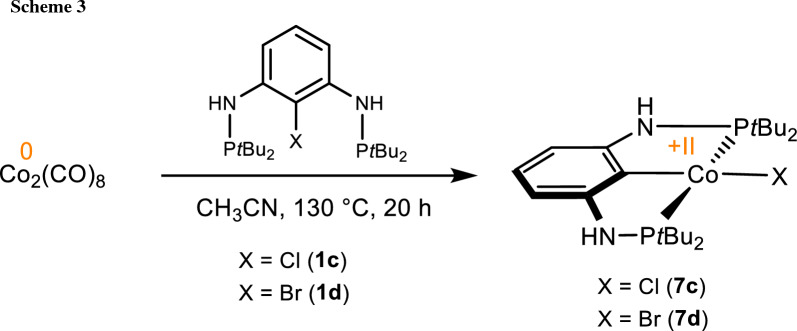

**Fig. 3:**
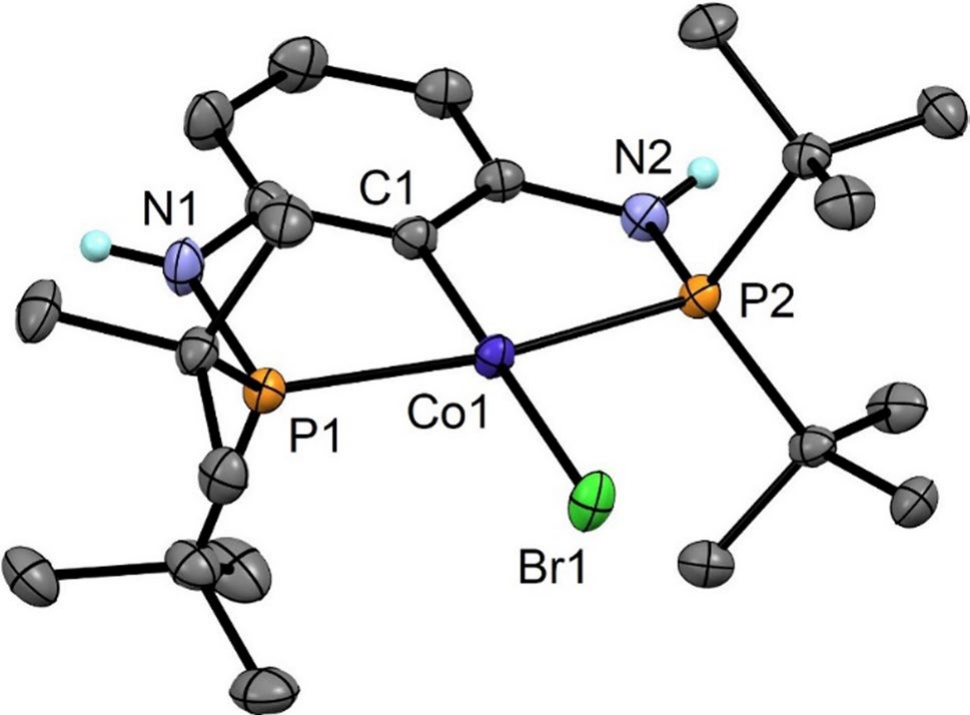
Structural view of [Co(PCP^NH^-*t*Bu)Br]⋅CH_3_CN (**7d**⋅CH_3_CN) showing 50% thermal ellipsoids (most H atoms and solvent omitted for clarity). Selected bond lengths (Å) and bond angles (deg): Co1-C1 1.930(2), Co1-P1 2.2393(7), Co1-P2 2.2332(7), Co1-Br1 2.3686(6), N1-HN1 0.85(2), P1-Co1-P2 165.22(3), C1-Co1-Br1 179.87(5)

The solid-state structure of **7d** was determined by X-ray diffraction. A representation of the molecule is given in Fig. 3 with selected metrical parameters provided in the captions. It has to be noted that structurally characterized square planar complexes of Co(II)-X are rare generally requiring strong-field ligands [31]. The molecular structure of **7d** shows the metal in a typical slightly distorted-square planar conformation. The C1-Co1-Br1 angles deviate slight from linearity being 179.87(5)°. The P(1)-Co1-P2 angle is 165.22(3)°.

With P(C-Br)P^CH2^-R (**2a**, **2b**) bearing CH_2_ linkers under solvothermal conditions at 110 °C or 130 °C a stoichiometric mixture of [Co(PCP^CH2^-*i*Pr)(CO)_2_] (**8**) and [Co(PCP^CH2^-*i*Pr)-Br_2_] (**10a**) or [Co(PCP^CH2^-*t*Bu)(CO)_2_] (**9**) and [Co(PCP^CH2^-*t*Bu)Br_2_] (**10b**) was obtained (Scheme 4).

**Figure:**
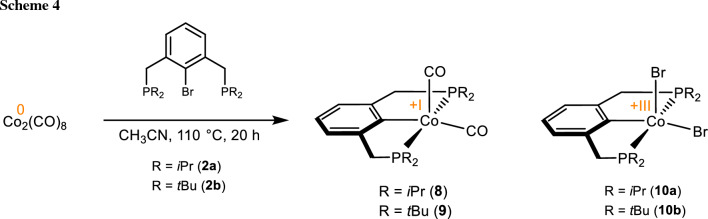

Complexes **8** and **9** display two strong absorption bands in the IR spectrum observed at 1966 and 1907 cm^−1^ and 1963 and 1904 cm^−1^, respectively, being characteristic for a *cis*-dicarbonyl arrangement. In the ^13^C{^1^H} NMR spectrum, the two CO ligands exhibit one low-field resonance at 210.3 and 212.1 ppm, while the *ipso*-carbons give rise to a triplet resonance at 169.8 ppm (*J*_*PC*_ = 15.8 Hz) and broad signal at 171.3 ppm.

**Fig. 4:**
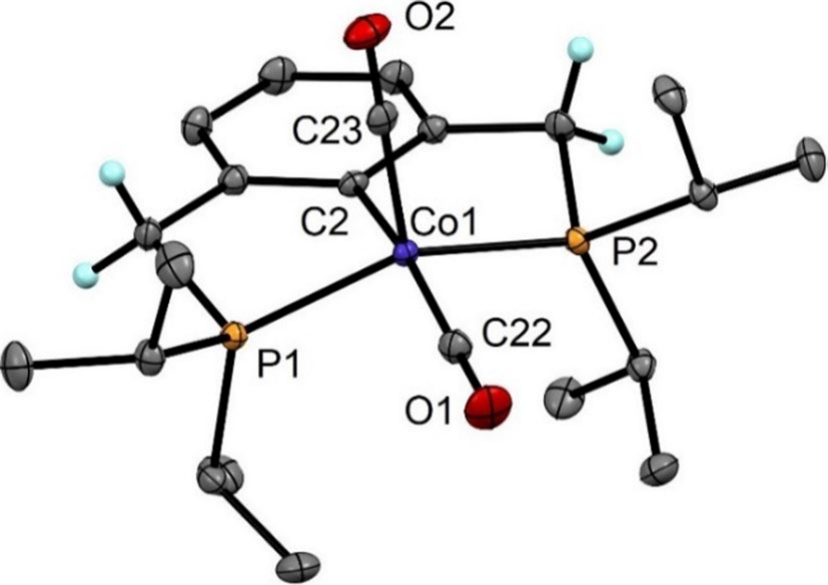
Structural view of [Co(PCP^CH2^-*i*Pr)(CO)_2_] (**8**) showing 50% ellipsoids (most H atoms omitted for clarity). Selected bond lengths (Å) and bond angles (deg): Co1-C2 2.013(1), Co1-P1 2.1942(6), Co1-P2 2.1919(7), Co1-C22 1.756(2), Co1-C23 1.794(2), C22-O1 1.167(3), C23-O2 1.148(2), P1-Co1-P2 144.83(2), C2-Co1-C22 164.75(8), C2-Co1-C23 91.38(6), C22-Co1-C23 103.87(8)

**Fig. 5:**
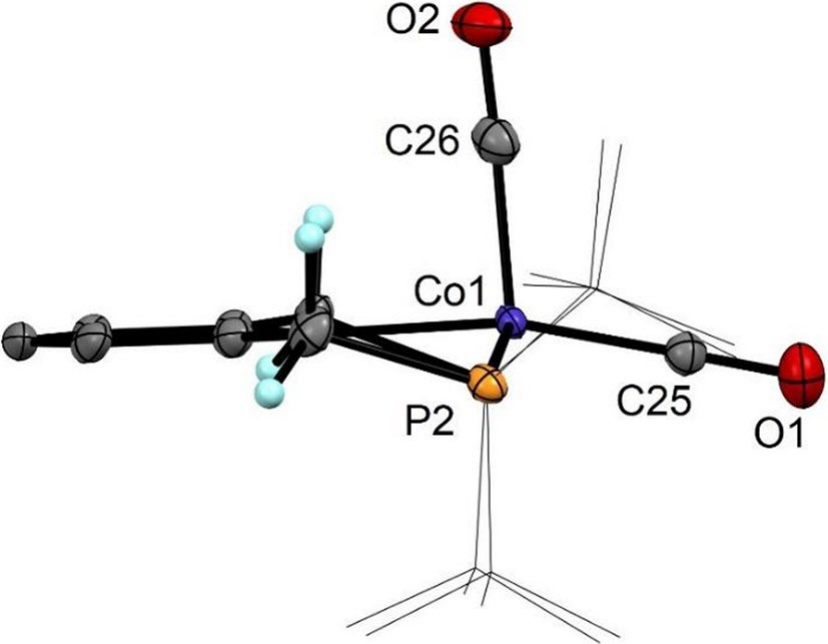
Structural view of [Co(PCP^CH2^-*t*Bu)(CO)_2_] (**9**) showing 50% ellipsoids (H atoms omitted for clarity). Selected bond lengths (Å) and bond angles (deg): Co1-C1 1.995(3), Co1-P1 2.2365(9), Co1-P2 2.2444(9), Co1-C25 1.748(3), Co1-C26 1.785(3), C25-O1 1.157(5), C26-O2 1.150(3), P1-Co1-P2 146.53(4), C1-Co1-C25 166.3(1), C1-Co1-C26 88.7(1), C25-Co1-C26 105.0(1)

In addition, the molecular structures of complexes **8** and **9** were determined by X-ray crystallography (Figs. 4 and 5). Selected bond distances are given in the captions. Both complexes adopt square pyramidal coordination geometries as also seen from the structural parameter τ_5_ being 0.33 and (τ_5_ = 0 indicates an ideal square pyramidal structure). Unlike the NH congener, the sp^3^ hybridized methylene spacer groups allow superior flexibility due to the lack of π-interaction with the aromatic backbone. Hence, the metal center and the ligand backbone are not aligned in a plane like in complex **4**. Table 1 provides an overview of selected crystallographic parameters of dicarbonyl Co(I) PCP pincer complexes.

**Table 1:** Selected metric parameters of some Co(I) PCP dicarbonyl complexes

Complex	τ_5_	Bond angles/deg		
		P1-Co-P2	C_ipso_-Co-CO_ap_	C_ipso_-Co-CO_bas_
[Co(PCP^NH^-*i*Pr)(CO)_2_] (**4**)	0.21	153.46(3)	110.5(1)	141.1(1)
[Co(PCP^CH2^-*i*Pr)(CO)_2_] (**8**)	0.33	144.83(2)	91.38(6)	164.8(1)
[Co(PCP^CH2^-*t*Bu)(CO)_2_] (**9**)	0.33	146.53(4)	88.7(1)	166.3(1)
[Co(PCP^O^-*i*Pr)(CO)_2_]^a^	0.32	155.02 (1)	114.95(5)	135.84(5)
[Co(PCP^O^-*t*Bu)(CO)_2_]^b^	0.08	152.28(2)	106.29(7)	147.76(7)

^a^Data obtained from Ref. [27]

^b^Data obtained from Ref. [24]

The dibromide-complexes **10a** and **10b** were characterized by HR-MS measurements as well as measurement of the magnetic susceptibility (CH_2_Cl_2_, benzene, Evans method). The solution magnetic moments of 3.0(8) and 3.1(4) μ_B_, respectively, are consistent with a d^6^ intermediate spin system corresponding to two unpaired electrons and an oxidation state of + III.

## Conclusion

The synthesis and characterization of a series of cobalt PCP pincer complexes in the formal oxidation states + I, + II, and + III is described. The new PCP cobalt complexes were obtained by treatment of [Co_2_(CO)_8_] with *ipso*-substituted P(C_ipso_-X)P^Y^ ligands (X = Br, Cl; R = *i*Pr, *t*Bu) bearing Y = NH and CH_2_ linkers under solvothermal conditions. Isopropyl containing phosphines display, regardless of the spacer groups and the reaction temperature, a disproportionation after oxidative addition of the C–X bond. PCP complexes bearing *t*Bu-phosphines show a temperature-dependent behavior. At 110 °C PCP-*t*Bu with NH spacers undergo disproportionation, whereas at 130 °C oxidative addition is accompanied by loss of all carbonyl ligands affording a coordinatively unsaturated Co(II) complex. Due to the increased flexibility of CH_2_ spacers as compared to NH linkers, [Co_2_(CO)_8_] disproportionates with PCP^CH2^-*t*Bu to Co(I) and Co(III) complexes.

## Experimental

All manipulations were performed under an inert atmosphere of argon by using Schlenk techniques or in an MBraun inert-gas glovebox. The solvents were purified according to standard procedures [32]. The deuterated solvents were purchased from Eurisotop SAS and dried over 4 Å molecular sieves. All starting materials are known compounds and were used as obtained from commercial resources. The ligands P(C-Br)-P^CH2^-*i*Pr (**2a**) [33] and P(C-Br)P^CH2^-*t*Bu (**2b**) [34] were prepared according to the literature. ^1^H, ^13^C{^1^H}, and ^31^P{^1^H} NMR spectra were recorded on Bruker AVANCE-250, AVANCE-400, and AVANCE-600 spectrometers^1^H and ^13^C{^1^H} NMR spectra were referenced internally to residual protio-solvent and solvent resonances, respectively, and are reported relative to tetramethylsilane (*δ* = 0 ppm). ^31^P{^1^H} NMR spectra were referenced externally to H_3_PO_4_ (85%) (*δ* = 0 ppm). Infrared spectra were recorded in attenuated total reflection (ATR) mode on a PerkinElmer Spectrum Two FT-IR spectrometer.

High resolution-accurate mass spectra were recorded on a hybrid Maxis Qq-aoTOF mass spectrometer (Bruker Daltonics, Bremen, Germany) fitted with an ESI source or an Agilent 6545 QTOF mass spectrometer equipped with an Agilent Dual AJS ESI ion source (Agilent Technologies, Santa Clara, CA, USA). Measured accurate mass data of the [M]^+^ ions for confirming calculated elemental compositions were typically within ± 5 ppm accuracy. The mass calibration was done with a commercial mixture of perfluorinated trialkyl-triazines (ES Tuning Mix, Agilent Technologies, Santa Clara, CA, USA).

### 2-Chloro-*N*,*N'*-bis(diisopropylphosphino)-1,3-diaminobenzene (P(C–Cl)P^NH^-*i*Pr, 1a, C_18_H_33_ClN_2_P_2_) 

2-Chlorobenzene-1,3-diamine (0.50 g, 3.5 mmol) and diisopropylethylamine (DIPEA) (1.11 g, 8.6 mmol) were added to a Schlenk flask and dissolved in dry toluene (10 cm^3^). Chloro(diisopropyl)phosphine (96%, 1.38 g, 9.0 mmol) was added dropwise to the reaction mixture while cooling with an ice bath, whereupon the mixture turned turbid. After complete addition, the mixture was stirred at 80 °C for 6 days. The reaction mixture was reduced to half its original volume and filtered through a pad of silica. After removing of all volatiles, **1a** was obtained as colorless oil. Yield: 1.06 g (80%); ^1^H NMR (600 MHz, CD_2_Cl_2_, 20 °C): *δ* = 6.82 (t,* J* = 8.2 Hz, 1H, CH), 6.69 (dd, *J* = 8.1, 3.3 Hz, 2H, CH), 4.25 (d*, J* = 10.3 Hz, 2H, N–H), 1.69 (m, 4H, C*H*(CH_3_)_2_), 0.98 (m, 24H, CH(C*H*_3_)_2_) ppm; ^13^C{^1^H} NMR (151 MHz, CD_2_Cl_2_, 20 °C): *δ* = 146.1 (d, *J* = 17.3 Hz, C-N), 127.43 (s, CH), 109.1 (s, C-Cl), 106.4 (d, *J* = 23.0 Hz, CH), 27.3 (d, *J* = 12.2 Hz, P-CH), 19.2 (d, *J* = 20.3 Hz, CH(*C*H_3_)_2_), 17.4 (d, *J* = 7.6 Hz, CH(*C*H_3_)_2_) ppm; ^31^P{^1^H} NMR (243 MHz, CD_2_Cl_2_, 20 °C): *δ* = 50.0 ppm; HR-MS (ESI^+^, CH_3_CN/MeOH + 1% H_2_O): *m/z* calcd for C_18_H_34_ClN_2_P_2_ ([M + H]^+^) 375.1880, found 375.1885.

### 2-Bromo-*N*,*N'*-bis(diisopropylphosphino)-1,3-diaminobenzene, P(C–Br)P^NH^-*i*Pr (1b, C_18_H_33_BrN_2_P_2_)

 2-Bromobenzene-1,3-diamine (0.40 g, 2.2 mmol) and DIPEA (1.11 g, 8.6 mmol) were added to a Schlenk flask and dissolved in toluene (10 cm^3^). Chloro(diisopropyl)phosphine (96%, 0.697 g, 4.4 mmol) was added dropwise to the reaction mixture while cooling with an ice bath, whereupon the mixture turned turbid. After complete addition, the mixture was stirred at 80 °C for 3 days. The reaction mixture was reduced to 10 cm3 and was filtered through a pad of celite. All volatiles were removed under reduced pressure resulting in the formation of an off-white viscous liquid. The oil was redissolved in *n*-pentane and filtered through a pad of silica. After removing all volatiles, **1b** was obtained as a colorless oil. Yield: 0.703 g (78%); ^**1**^H NMR (400 MHz, CD_2_Cl_2_, 20 °C): *δ* = 6.97 (d, *J* = 8.2 Hz, 1H, CH), 6.81 (m, 2H, CH), 4.42 (d, *J* = 10.2 Hz, 2H, N-H), 1.82 (m, 4H, C*H*(CH_3_)_2_), 1.11 (m, 24H, CH(C*H*_3_)_2_) ppm; ^13^C{^1^H} NMR (101 MHz, CD_2_Cl_2_, 20 °C): *δ* = 147.2 (d,* J* = 17.3 Hz, C-N), 128.2 (s, CH), 106.7 (d,* J* = 23.3 Hz, CH), 103.0 (s, C-Br), 27.3 (d,* J* = 12.3 Hz, P-CH), 19.2 (d,* J* = 20.3 Hz, CH(*C*H_3_)_2_), 17.5 (d, *J* = 7.7 Hz, CH(*C*H_3_)_2_) ppm; ^31^P{^1^H} NMR (162 MHz, CD_2_Cl_2_, 20 °C): *δ* = 50.7 ppm; HR-MS (ESI^+^, CH_3_CN/MeOH + 1% H_2_O): *m/z* calcd for C_18_H_34_BrN_2_P_2_ ([M + H]^+^) 419.1375, found 419.1375.

### 2-Chloro-*N*,*N'*-bis(di-*tert*-butylphosphino)-1,3-diaminobenzene, P(C-Cl)P^NH^-*t*Bu (1c, C_22_H_41_ClN_2_P_2_) 

2-Chlorobenzene-1,3-diamine (0.50 g, 3.5 mmol) and DIPEA (0.93 g, 7.2 mmol) were added to a Schlenk flask and dissolved in toluene (15 cm^3^). Chlorodi(*tert-*butyl)phosphine (96%, 1.30 g, 7.2 mmol) was added dropwise while cooling with an ice bath. Then NaH (181 mg, 7.7 mmol), suspended in THF (10 cm^3^), was submitted to the mixture, which was stirred at 80 °C for 24 h. All volatiles were removed under reduced pressure resulting in the formation of a beige solid which was redissolved in *n*-pentane, filtered through a pad of silica and washed with *n*-pentane. After removing all volatiles under reduced pressure at 80 °C, **1c** was afforded as a colorless solid with a yield of 1.048 g (69%). ^1^H NMR (600 MHz, CD_2_Cl_2_, 20 °C): *δ* = 6.91 (t,* J* = 8.1 Hz, 1H, CH), 6.79 (dd, *J* = 8.1, 3.5 Hz, 2H, CH), 4.70 (d,* J* = 10.2 Hz, 2H, N–H), 1.14 (d, *J* = 11.9 Hz, 36H, C(CH_3_)_3_) ppm; ^13^C{^1^H} NMR (151 MHz, CD_2_Cl_2_, 20 °C): *δ* = 146.3 (d,* J* = 18.2 Hz, C-N), 127.41 (s, CH), 108.7 (s, C–Cl), 106.4 (d,* J* = 22.7 Hz, CH), 34.7 (d, *J* = 20.3 Hz, *C*(CH_3_)_3_), 28.4 (d,* J* = 15.4 Hz, C(*C*H_3_)_3_) ppm; ^31^P{^1^H} NMR (243 MHz, CD_2_Cl_2_, 20 °C): *δ* = 59.8 ppm; HR-MS (ESI^+^, CH_3_CN/MeOH + 1% H_2_O): *m/z* calcd for C_22_H_42_ClN_2_P_2_ ([M + H]^+^) 431.2506, found 431.2512.

### 2-Bromo-*N*,*N'*-bis(di-*tert*-butylphosphino)-1,3-diaminobenzene, P(C-Br)P^NH^-*t*Bu (1d, C_22_H_41_BrN_2_P_2_) 

2-Bromobenzene-1,3-diamine (0.42 g, 2.2 mmol) and DIPEA (0.58 g, 4.5 mmol) were added to a Schlenk flask and dissolved in toluene (10 cm^3^). Chlorodi(*tert-*butyl)phosphine (96%, 0.866 g, 4.6 mmol) was added dropwise while cooling with an ice bath. Then NaH (116 mg, 4.8 mmol), suspended in THF (10 cm^3^), was submitted to the mixture, which was stirred at 80 °C for 24 h. All volatiles were removed under reduced pressure resulting in the formation of a beige solid which was redissolved in *n*-pentane, filtered through a pad of silica and washed with *n*-pentane. After removing all volatiles under reduced pressure at 80 °C, **1d** was afforded as a colorless solid. Yield: 0.586 g (56%); ^1^H NMR (400 MHz, CD_2_Cl_2_, 20 °C): *δ* = 6.94 (t, *J* = 8.1 Hz, 1H, CH), 6.79 (dd, *J* = 8.1, 3.5 Hz, 2H, CH), 4.75 (d, *J* = 10.1 Hz, 2H, N–H), 1.15 (d,* J* = 11.8 Hz, 36 H, C(CH_3_)_3_) ppm; ^13^C{^1^H} NMR (101 MHz, CD_2_Cl_2_, 20 °C): *δ* = 147.4 (d, *J* = 17.8 Hz, C-N), 128.1 (s, CH), 106.7 (d, *J* = 23.3 Hz, CH), 102.6 (s, C–Br), 34.7 (d, *J* = 20.2 Hz, C(CH_3_)_3_), 28.4 (d, *J* = 15.2 Hz, C(CH_3_)_3_) ppm; ^31^P{^1^H} NMR (162 MHz, CD_2_Cl_2_, 20 °C): *δ* = 60.5 ppm; HR-MS (ESI^+^, CH_3_CN/MeOH + 1% H_2_O): *m/z* calcd for C_22_H_42_BrN_2_P_2_ ([M + H]^+^) 475.2000, found 475.2003.

### [2,6-Bis[[bis(1-methylethyl)phosphino-κP]-amino]phenyl-κC](dicarbonyl)cobalt(I), [Co(PCP^NH^-*i*Pr)(CO)_2_] (4, C_20_H_33_CoN_2_O_2_P_2_) 

A solution of **1a** (50 mg, 0.13 mmol) or **1b** (50 mg, 0.12 mmol) and [Co_2_(CO)_8_] (0.5 equiv.) in acetonitrile (4 cm^3^) was put into a microwave vial and stirred at 110 °C for 20 h, whereupon the color of the reaction mixture changed from orange-red to green. The mixture was transferred into a Schlenk flask and all volatiles were removed under reduced pressure. The obtained residue was extracted with *n*-pentane and filtered through a syringe filter (PTFE, 0.2 µm). After removing all volatiles in vacuo, **4** could be isolated as a yellow solid with a yield of 23 mg (43%). Single crystals were obtained from slow evaporation of the solvent from a saturated solution of **4** in *n*-pentane at − 20 °C. ^1^H NMR (600 MHz, CD_2_Cl_2_, 20 °C): *δ* = 6.54 (t, *J* = 7.6 Hz, 1H, CH), 6.03 (d, *J* = 7.6 Hz, 2H, CH), 4.18 (s, 2H, NH), 2.33 (m, 4H, C*H*(CH_3_)_2_), 1.23 (m, 24H, CH(C*H*_3_)_2_) ppm; ^13^C{^1^H} NMR (151 MHz, CD_2_Cl_2_, 20 °C): *δ* = 207.4 (br, CO), 155.7 (t, *J* = 12.9 Hz, C-N), 133.1 (t, *J* = 17.9 Hz, C_ipso_), 124.1 (s, CH), 100.6 (t, *J* = 7.0 Hz, CH), 31.5 (t, *J* = 12.9 Hz, *C*H(CH_3_)_2_), 18.0 (d, *J* = 92.7 Hz, CH(*C*H_3_)_2_ ppm; ^31^P{^1^H} NMR (243 MHz, CD_2_Cl_2_, 20 °C): *δ* = 158.5 ppm; IR (ATR):  = 1896 (ν_CO_), 1956 (ν_CO_) cm^−1^; HR-MS (ESI^+^, CH_3_CN/MeOH + 1% H_2_O): *m/z* calc for C_19_H_33_CoN_2_OP_2_ ([M-CO]^+^) 426.1388, found 426.1401.

### [2,6-Bis[[bis(1-methylethyl)phosphino-κP]amino]-phenyl-κC](dichloro)cobalt(III), [Co(PCP^NH^-*i*Pr)Cl_2_] (6a, C_18_H_32_Cl_2_CoN_2_P_2_) 

A solution of **1a** (50 mg, 0.13 mmol) and [Co_2_(CO)_8_] (22 mg, 0.07 mmol) in acetonitrile (4 cm^3^) was put into a microwave vial and stirred at 110 °C for 20 h, whereupon the color of the reaction mixture changed from orange-red to green. The mixture was transferred into a Schlenk flask and all volatiles were removed under reduced pressure. The obtained residue was extracted with *n*-pentane to afford **6a** as a dark green solid with a yield of 26 mg (42%). HR-MS (ESI^+^, CH_3_CN/MeOH + 1% H_2_O): *m/z* calc for C_18_H_33_CoN_2_P_2_ ([M-2Cl + H]^+^) 399.1524, found 399.1534; *µ*_eff_ = 3.1(8) µ_B_ (benzene, Evans method).

### [2,6-Bis[[bis(1-methylethyl)phosphino-κP]amino]-phenyl-κC](dibromo)cobalt(III), [Co(PCP^NH^-*i*Pr)Br_2_] (6b, C_18_H_32_Br_2_CoN_2_P_2_) 

A solution of **1b** (50 mg, 0.12 mmol) and [Co_2_(CO)_8_] (22 mg, 0.07 mmol) in acetonitrile (4 cm^3^) was put into a microwave vial and stirred at 110 °C for 20 h, whereupon the color of the reaction mixture changed from orange-red to green. The mixture was transferred into a Schlenk flask and all volatiles were removed under reduced pressure. The obtained residue was extracted with *n*-pentane in order to remove **4**, to afford **6b** as a dark green–brown solid with a yield of 31 mg (46%). HR-MS (ESI^+^, CH_3_CN/MeOH + 1% H_2_O): *m/z* calc for C_18_H_33_CoN_2_P_2_ ([M-2Br + H]^+^) 399.1524, found 399.1528; *µ*_eff_ = 3.2(3) µ_B_ (benzene, Evans method).

### [2,6-Bis[[bis(1,1-dimethylethyl)phosphino-κP]-amino]phenyl-κC](dichloro)cobalt(III), [Co(PCP^NH^-*t*Bu)Cl_2_] (6c, C_22_H_41_Cl_2_CoN_2_P_2_) 

A solution of **1c** (50 mg, 0.12 mmol) and [Co_2_(CO)_8_] (20 mg, 0.06 mmol) in acetonitrile (4 cm^3^) was put into a microwave vial and was stirred at 110 °C for 20 h, whereupon the color of the reaction mixture changed from orange-red to green. The mixture was transferred into a Schlenk flask and all volatiles were removed under reduced pressure. The obtained residue was extracted with *n*-pentane. After removing all volatiles in vacuo **6**c was obtained as a yellow solid with a yield of 24 mg (39%). HR-MS (ESI^+^, CH_3_CN/MeOH + 1% H_2_O): *m/z* calcd for C_22_H_41_ClCoN_2_P_2_ ([M-Cl]^+^) 489.1759, found 489.1762; *µ*_eff_ = 3.2(9) µ_B_ (THF, Evans method).

### [2,6-Bis[[bis(1,1-dimethylethyl)phosphino-κP]-amino]phenyl-κC](dibromo)cobalt(III), [Co(PCP^NH^**-***t*Bu)Br_2_] (6d, C_22_H_41_Br_2_CoN_2_P_2_)

A solution of **1d** (50 mg, 0.11 mmol) and [Co_2_(CO)_8_] (18 mg, 0.05 mmol) in acetonitrile (4 cm^3^) was put into a microwave vial and was stirred at 110 °C for 20 h, whereupon the color of the reaction mixture changed from orange-red to green. The mixture was transferred into a Schlenk flask and all volatiles were removed under reduced pressure. The obtained residue was extracted with *n*-pentane and filtered through a syringe filter (PTFE, 0.2 µm). After removing all volatiles in vacuo, **6d** was obtained as a yellow solid with a yield of 24 mg (38%). HR-MS (ESI^+^, CH_3_CN/MeOH + 1% H_2_O): *m/z* calcd for C_22_H_41_BrCoN_2_P_2_ ([M-Br]^+^) 533.1264, found 533.1254; *µ*_eff_ = 3.3(5) µ_B_ (THF, Evans method).

### [2,6-Bis[[bis(1,1-dimethylethyl)phosphino-κP]-amino]phenyl-κC](chloro)cobalt(II), [Co(PCP^NH^-*t*Bu)Cl] (7c, C_22_H_41_ClCoN_2_P_2_)

A solution of **1c** (50 mg, 0.13 mmol) and [Co_2_(CO)_8_] (23 mg, 0.07 mmol) in acetonitrile (4 cm^3^) was put into a microwave vial and was stirred at 130 °C for 20 h, whereupon the color of the reaction mixture changed from yellow to red. The mixture was transferred into a Schlenk flask and all volatiles were removed under reduced pressure. The obtained residue was extracted with *n*-pentane to remove any impurities and **7c** was afforded as an orange-red solid with a yield of 51 mg (89%). HR-MS (ESI^+^, CH_3_CN/MeOH + 1% H_2_O): *m/z* calcd for C_22_H_41_CoN_2_P_2_ ([M-Cl]^+^) 454.2065, found 454.2079; *µ*_eff_ = 1.8(2) µ_B_ (CH_2_Cl_2_, Evans method).

### [2,6-Bis[[bis(1,1-dimethylethyl)phosphino-κP]-amino]phenyl-κC](bromo)cobalt(II), [Co(PCP^NH^-*t*Bu)Br] (7d, C_22_H_41_BrCoN_2_P_2_) 

A solution of **1d** (50 mg, 0.11 mmol) and [Co_2_(CO)_8_] (18 mg, 0.05 mmol) in acetonitrile (4 cm^3^) was put into a microwave vial and was stirred at 130 °C for 20 h, whereupon the color of the reaction mixture changed from yellow to red. The mixture was transferred into a Schlenk flask and all volatiles were removed under reduced pressure. The obtained residue was extracted with *n*-pentane to remove any impurities and **7d** was afforded as an orange-red solid with a yield of 53 mg (91%). HR-MS (ESI^+^, CH_3_CN/MeOH + 1% H_2_O): *m/z* calcd for C_22_H_41_CoN_2_P_2_ ([M-Br]^+^) 454.2065, found 454.2074; *µ*_eff_ = 1.8(3) µ_B_ (benzene, Evans method).

### [2,6-Bis[[bis(1-methylethyl)phosphino-κP]methyl]-phenyl-κC](dicarbonyl)cobalt(I), [Co(PCP^CH2^-***i***Pr)(CO)_2_] (8, C_22_H_35_CoO_**2**_P_2_) 

A solution of **2a** (50 mg, 0.12 mmol) and [Co_2_(CO)_8_] (21 mg, 0.06 mmol) in toluene (4 cm^3^) was put into a microwave vial and was stirred at 120 °C for 16 h, whereupon a green-yellow solution and a green precipitate formed. The solution was transferred into a Schlenk flask and all volatiles were removed under reduced pressure. The residue was extracted with benzene and all volatiles were removed in vacuo affording **8** as a yellow solid with a yield of 24 mg (44%). ^**1**^H NMR (600 MHz, C_6_D_6_, 20 °C): *δ* = 6.96 (s, 3H, CH), 3.03 (m, 4H, P-CH_2_), 1.93 (m, 4H, C*H*(CH_3_)_2_), 1.14 (app q, *J* = 7.0 Hz, *J* = 7.8 Hz, 12H, CH(C*H*_3_)_2_), 0.96 (app q, *J* = 7.0 Hz, *J* = 7.0 Hz, 12H, CH(C*H*_3_)_2_) ppm; ^13^C{^1^H} NMR (151 MHz, C_6_D_6_, 20 °C): *δ* = 210.3 (br, CO), 169.8 (t, *J* = 15.8 Hz, Co-C_ipso_), 147.2 (t, *J* = 11.5 Hz, P-CH_2_-*C*_Ar_), 123.0 (s, CH), 121.7 (t, *J* = 8.7 Hz, CH), 39.0 (dd, *J* = 15.0, 12.5 Hz, P-CH_2_), 27.3 (t, *J* = 9.8 Hz, *C*H(CH_3_)_2_), 18.7 (d, *J* = 18.4 Hz, CH(*C*H_3_)_2_) ppm; ^31^P{^1^H} NMR (243 MHz, C_6_D_6_, 20 °C): *δ* = 102.9 ppm; IR (ATR):  = 1966 (ν_CO_), 1907 (ν_CO_) cm^−1^; HR-MS (ESI^+^, CH_3_CN/MeOH + 1% H_2_O): *m/z* calcd for C_21_H_35_CoOP_2_ ([M-CO]^+^) 424.1489, found 424.1501.

### [2,6-Bis[[bis(1,1-dimethylethyl)phosphino-κP]methyl]-phenyl-κC](dicarbonyl)cobalt(I), [Co(PCP^CH2^-***t***Bu)(CO)_2_] (9, C_26_H_43_CoO_**2**_P_2_) 

A solution of **2b** (50 mg, 0.11 mmol) and [Co_2_(CO)_8_] (20 mg, 0.06 mmol) in toluene (4 cm^3^) was put into a microwave vial and was stirred at 110 °C for 20 h, whereupon the color of the reaction mixture changed from dark orange to green. The mixture was transferred into a Schlenk flask and all volatiles were removed under reduced pressure affording a green solid. The residue was extracted with *n*-pentane and after removing all volatiles in vacuo, **9** was obtained as a light brown solid with a yield of 23 mg (43%). ^1^H NMR (400 MHz, CD_2_Cl_2_, 20 °C): *δ* = 6.81 (d, *J* = 7.3 Hz, 2H, CH), 6.66 (t, *J* = 7.3 Hz, 1H, CH), 3.36 (m, 4H, P-CH_2_), 1.35 (m, 36H, C(CH_3_)_3_) ppm; ^13^C{^1^H} NMR (101 MHz, CD_2_Cl_2_, 20 °C): *δ* = 212.1 (br, CO), 171.3 (br, Co-C_ipso_), 148.4 (t, *J* = 11.8 Hz, P-CH_2_-*C*_Ar_), 122.4 (s, CH), 120.8 (t, *J* = 8.5 Hz, CH), 38.8 (dd, *J* = 12.3, 10.5 Hz, P-CH_2_), 37.7 (t, *J* = 5.2 Hz, *C*(CH_3_)_3_), 30.1 (t, *J* = 2.2 Hz, C(*C*H_3_)_3_) ppm; ^31^P{^1^H} NMR (162 MHz, CD_2_Cl_2_, 20 °C): *δ* = 118.0 ppm; IR (ATR):  = 1962 (ν_CO_), 1904 (ν_CO_) cm^−1^; HR-MS (ESI^+^, CH_3_CN/MeOH + 1% H_2_O): *m/z* calcd for C_25_H_43_CoOP_2_ ([M-CO]^+^) 480.2115, found 480.2103.

### [2,6-Bis[[bis(1-methylethyl)phosphino-κP]methyl]-phenyl-κC](dibromo)cobalt(III), [Co(PCP^CH2^-*i*Pr)Br_2_] (10a, C_20_H_35_Br_2_CoP_2_) 

A solution of **2a** (50 mg, 0.12 mmol) and [Co_2_(CO)_8_] (22 mg, 0.07 mmol) in acetonitrile (4 cm^3^) was put into a microwave vial and stirred at 110 °C for 20 h, whereupon the color of the reaction mixture changed from orange-red to green. The mixture was transferred into a Schlenk flask and all volatiles were removed under reduced pressure. The obtained residue was extracted with *n*-pentane to afford **10a** as a dark green solid with a yield of 30 mg (45%). HR-MS (ESI^+^, CH_3_CN/MeOH + 1% H_2_O): *m/z* calc for C_20_H_35_CoP_2_ ([M-2Br]^+^) 396.1539, found 396.1541; *µ*_eff_ = 3.0(8) µ_B_ (CH_2_Cl_2_, Evans method).

### [2,6-Bis[[bis(1,1-dimethylethyl)phosphino-κP]methyl]phenyl-κC](dibromo)cobalt(III), [Co(PCP^CH2^-*t*Bu)Br_2_] (10b, C_24_H_43_Br_2_CoP_2_) 

A solution of **2b** (50 mg, 0.11 mmol) and Co_2_(CO)_8_ (20 mg, 0.06 mmol) in acetonitrile (4 cm^3^) was put into a microwave vial and stirred at 110 °C for 20 h, whereupon the color of the reaction mixture changed from dark orange to green. The mixture was transferred into a Schlenk flask and all volatiles were removed under reduced pressure. The obtained residue was extracted with *n*-pentane to afford **10b** as a dark green solid with a yield of 29 mg (45%). HR-MS (ESI^+^, CH_3_CN/MeOH + 1% H_2_O): *m/z* calc for C_24_H_43_CoP_2_ ([M-2Br]^+^) 452.2166, found 452.2157; *µ*_eff_ = 3.1(3) µ_B_ (benzene, Evans method).

### X-ray structure determination

X-ray diffraction data for **4**, **10b**‧CH_3_CN, **13a**, and **13b** (CCDC 2176522–2176525) were collected at *T* = 100 K in a dry stream of nitrogen on a Bruker Kappa APEX II diffractometer system using graphite-monochromatized Mo-*K*α radiation (*λ* = 0.71073 Å) and fine sliced *φ*- and *ω****-***scans. Data were reduced to intensity values with SAINT and an absorption correction was applied with the multi-scan approach implemented in SADABS [35]. The structures were solved by the dual space method implemented in SHELXT [36] and refined against *F*^2^ with SHELXL [37]. Non-hydrogen atoms were refined with anisotropic displacement parameters. The H atoms were placed in calculated positions and thereafter refined as riding on the parent C atoms. Molecular graphics were generated with the program MERCURY [38].

### Calculations

Calculations were performed using the Gaussian 09 software package [39], and the OPBE functional [40–45] without symmetry constraints, the Stuttgart/Dresden ECP (SDD) basis set to describe the electrons of the cobalt atom and a standard 6-31G** basis for all other atoms as already previously described [46].

### Electronic supplementary material

Below is the link to the electronic supplementary material.

Supplementary file1 (CIF 7401 kb)

Supplementary file2 (PDF 1501 kb)

## Data Availability

All relevant data are included in the manuscript.
