# Supplementary material for: Solvothermal synthesis of cobalt PCP pincer complexes from [Co2(CO)8]
Source: Monatsh Chem. 2023 Sep 9;154(11):1253–62. doi: 10.1007/s00706-023-03123-x (PMC10620272; doi:10.1007/s00706-023-03123-x)

Solvothermal synthesis of cobalt PCP pincer complexes from $[Co_2(CO)_8]$

Heiko Schratzberger[1] ● Daniel Himmelbauer[1] ● Wolfgang Eder[1] ● Michael Weiser[1] ● Berthold Stöger[2] ● Karl Kirchner[1] *

[1] Institute of Applied Synthetic Chemistry, Vienna University of Technology, Getreidemarkt 9/163-AC, 1060 Wien, Austria.

[2] X-Ray Center, Vienna University of Technology, Getreidemarkt 9/163-AC, 1060 Wien, Austria.

Supporting Information

NMR spectra

^{1}H-NMR (600 MHz, CD_2Cl_2, 20 °C): PCP^{NH}-*i*Pr-Cl (**1a**)

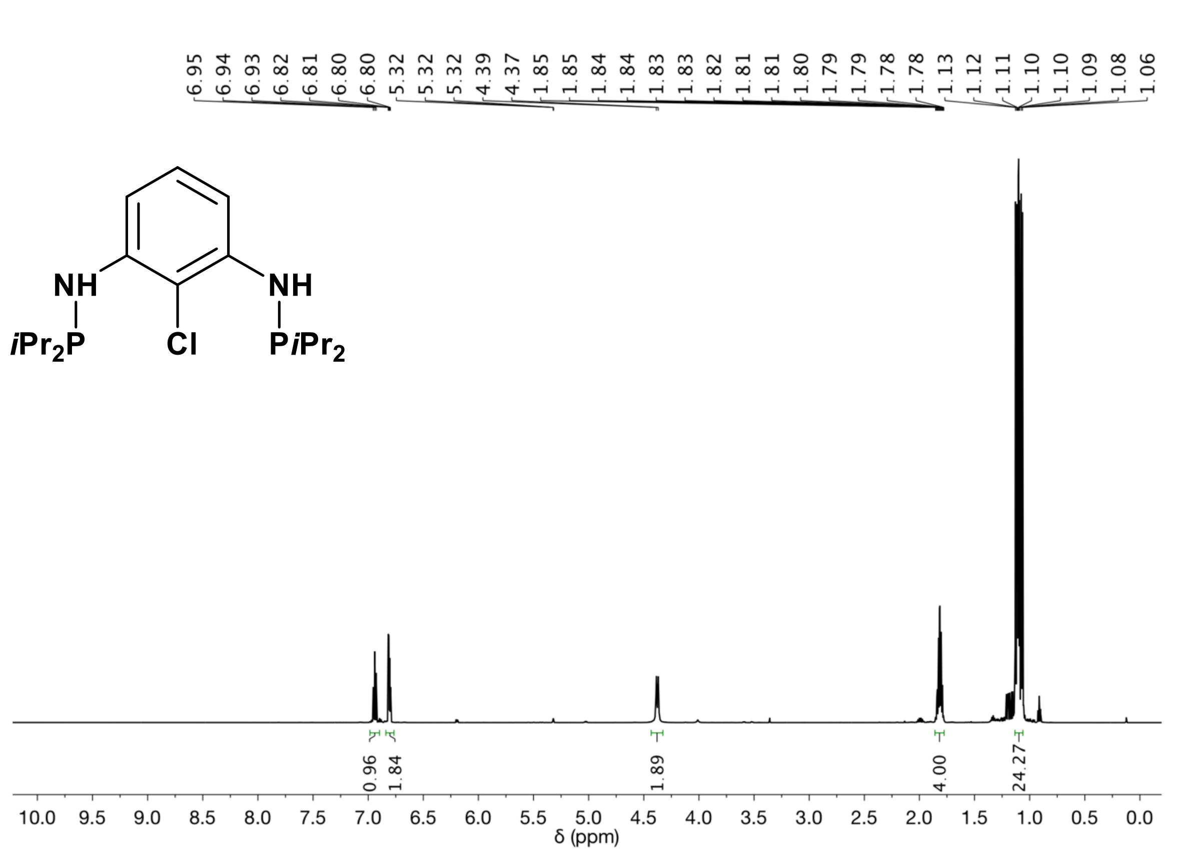

^{13}C{^{1}H}-NMR (151 MHz, CD_2Cl_2, 20 °C): PCP-NH-*i*Pr-Cl (**1a**)

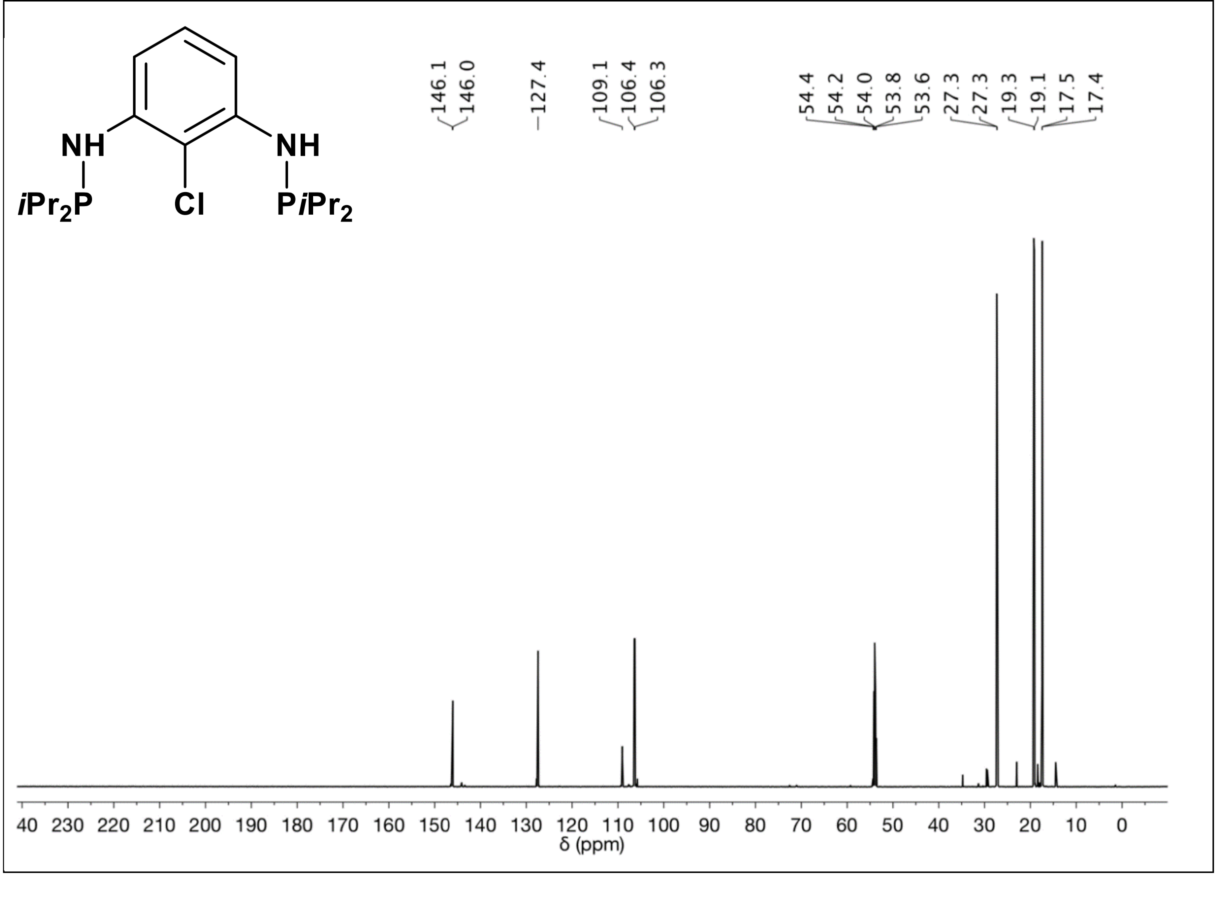

$^{31}P\{^{1}H\}$-NMR (243 MHz, CD_2Cl_2, 20 °C): PCP-NH-*i*Pr-Cl (**1a**)

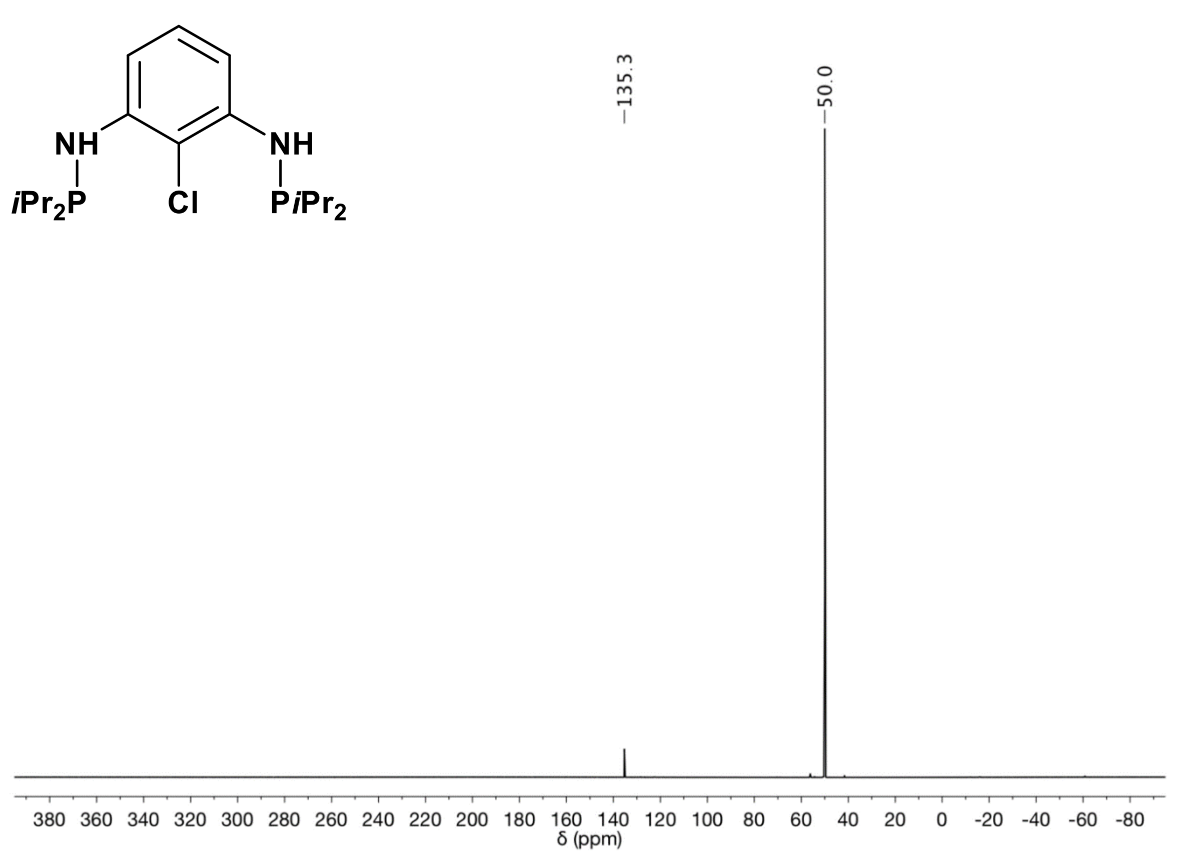

^{1}H-NMR (400 MHz, CD_2Cl_2, 20 °C): PCP^{NH}-*i*Pr-Br (**1b**)

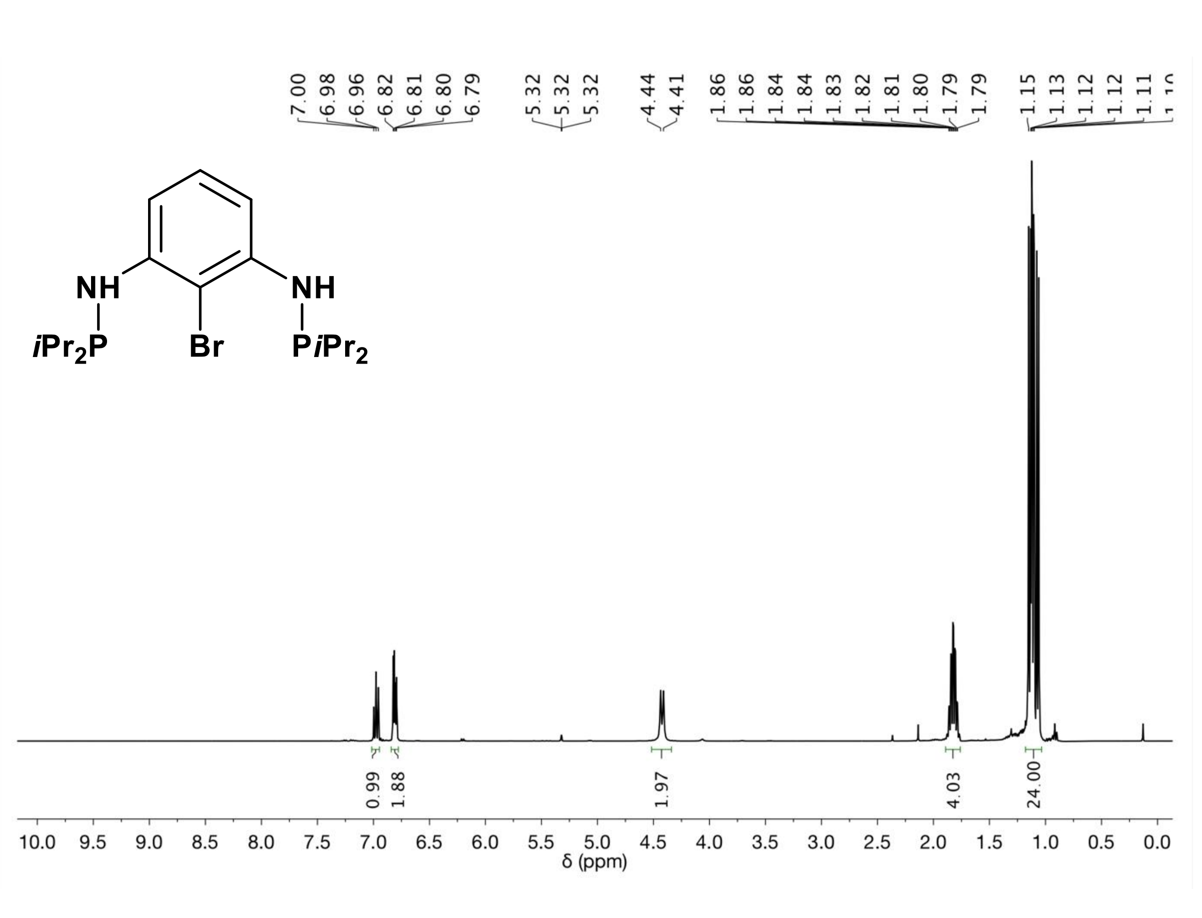

$^{13}C\{^{1}H\}$-NMR (400 MHz, CD_2Cl_2, 20 °C): PCP^{NH}-*i*Pr-Br (**1b**)

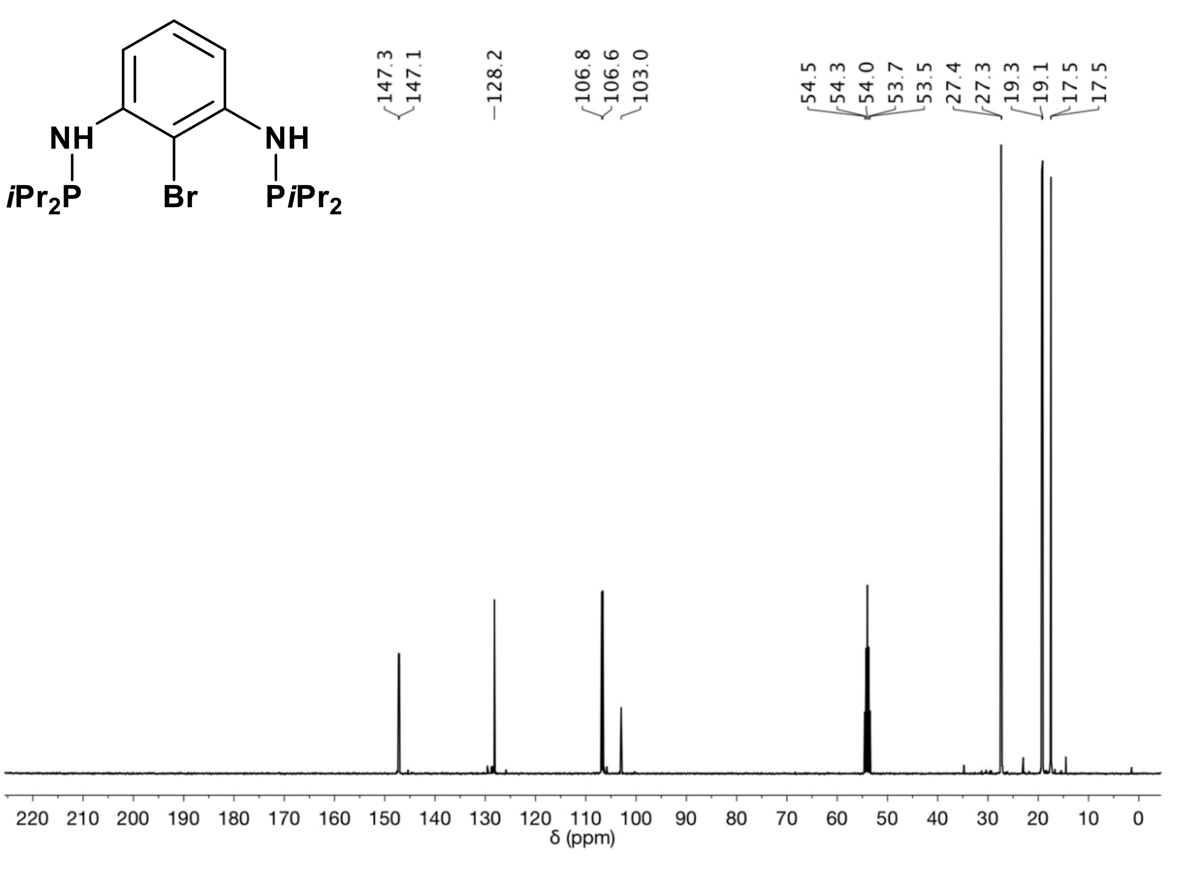

$^{31}P\{^{1}H\}$-NMR (400 MHz, CD_2Cl_2, 20 °C): PCP^{NH}-*i*Pr-Br (**1b**)

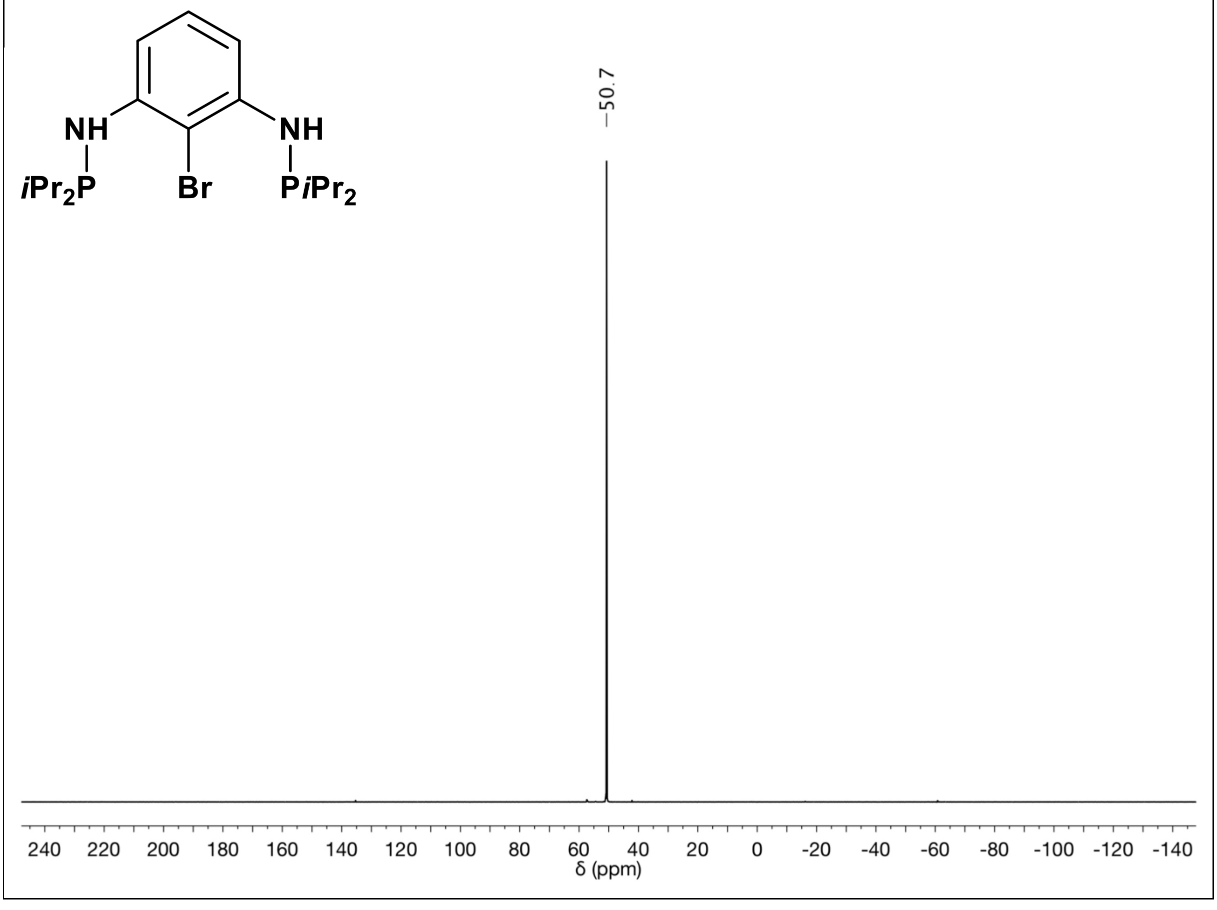

^{1}H-NMR (600 MHz, CD_2Cl_2, 20 °C): PCP^{NH}-*t*Bu-Cl (**1c**)

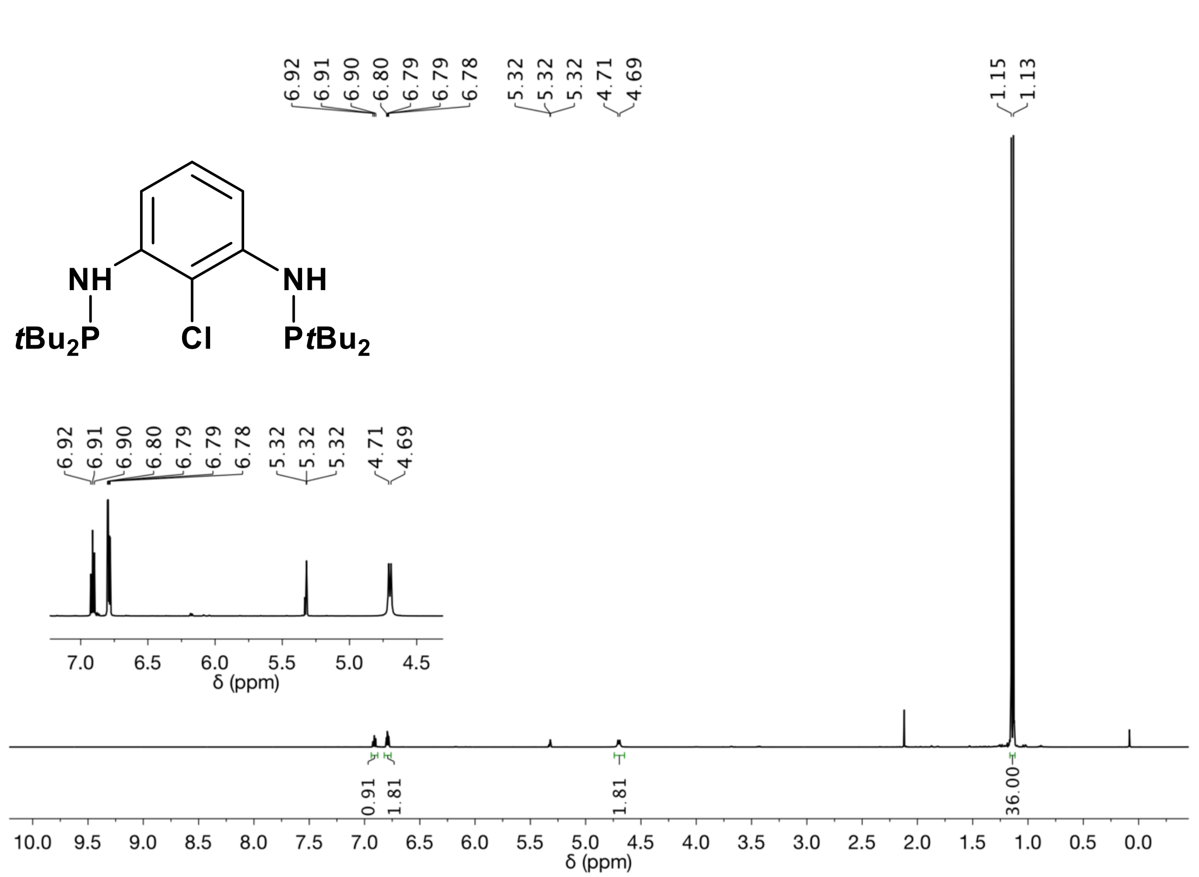

^{13}C{^{1}H}-NMR (151 MHz, CD_2Cl_2, 20 °C): PCP^{NH}-*t*Bu-Cl (**1c**)

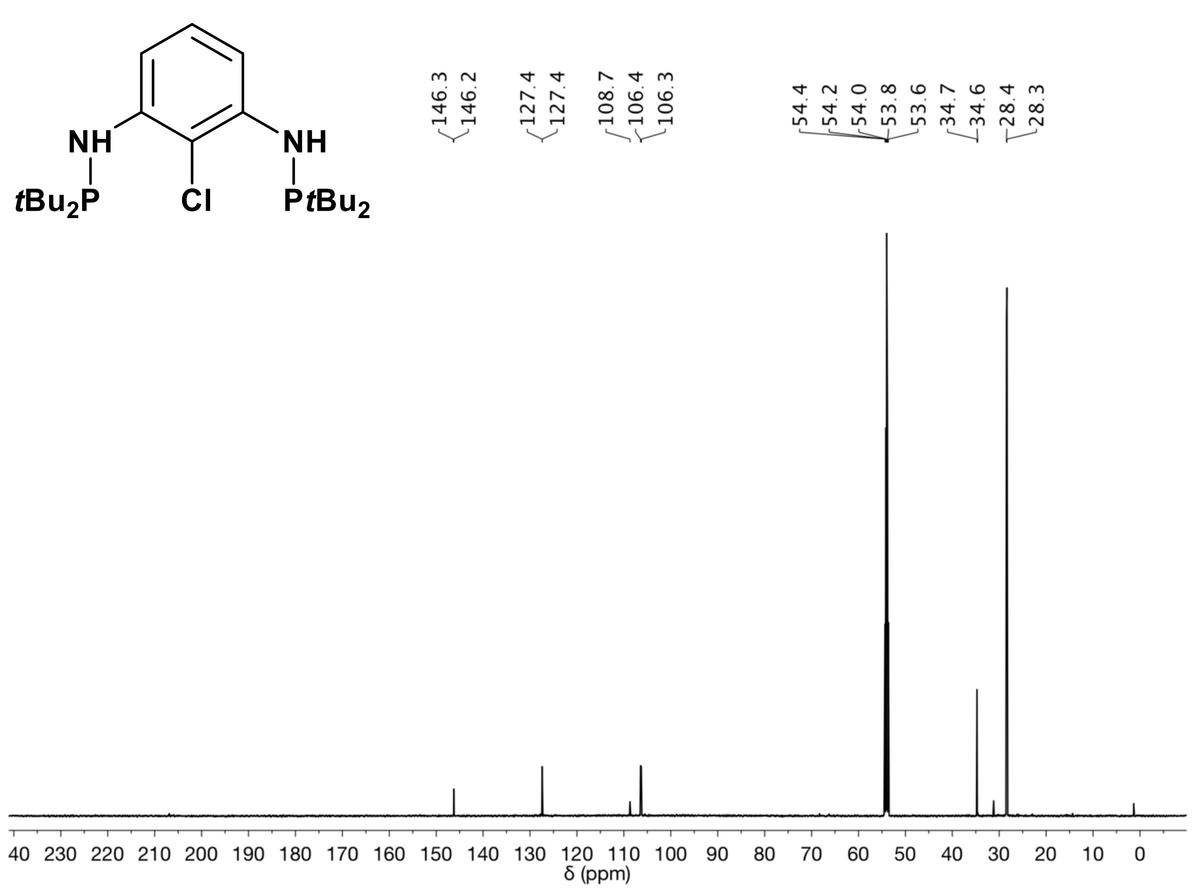

$^{31}P\{^{1}H\}$-NMR (243 MHz, CD_2Cl_2, 20 °C): PCP^{NH}-*t*Bu-Cl (**1c**)

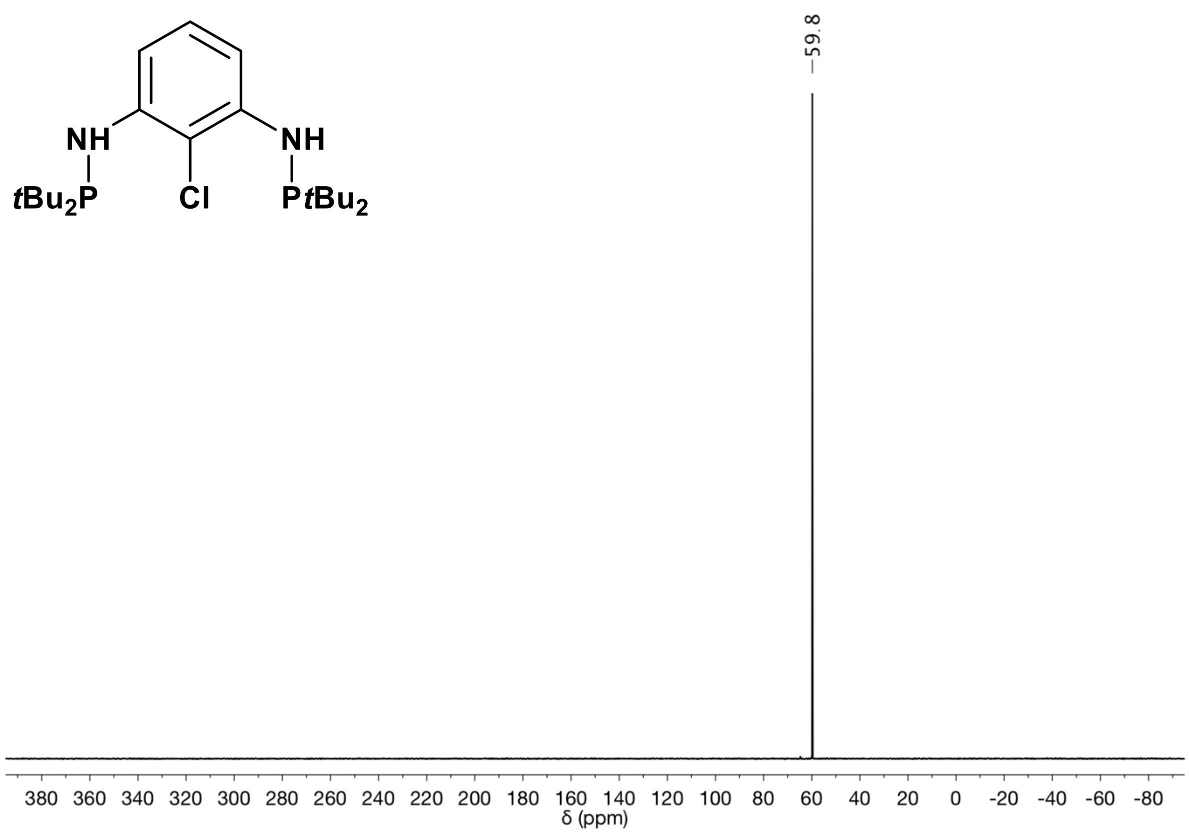

^{1}H-NMR (400 MHz, CD_2Cl_2, 20 °C): PCP^{NH}-*t*Bu-Br (**1d**)

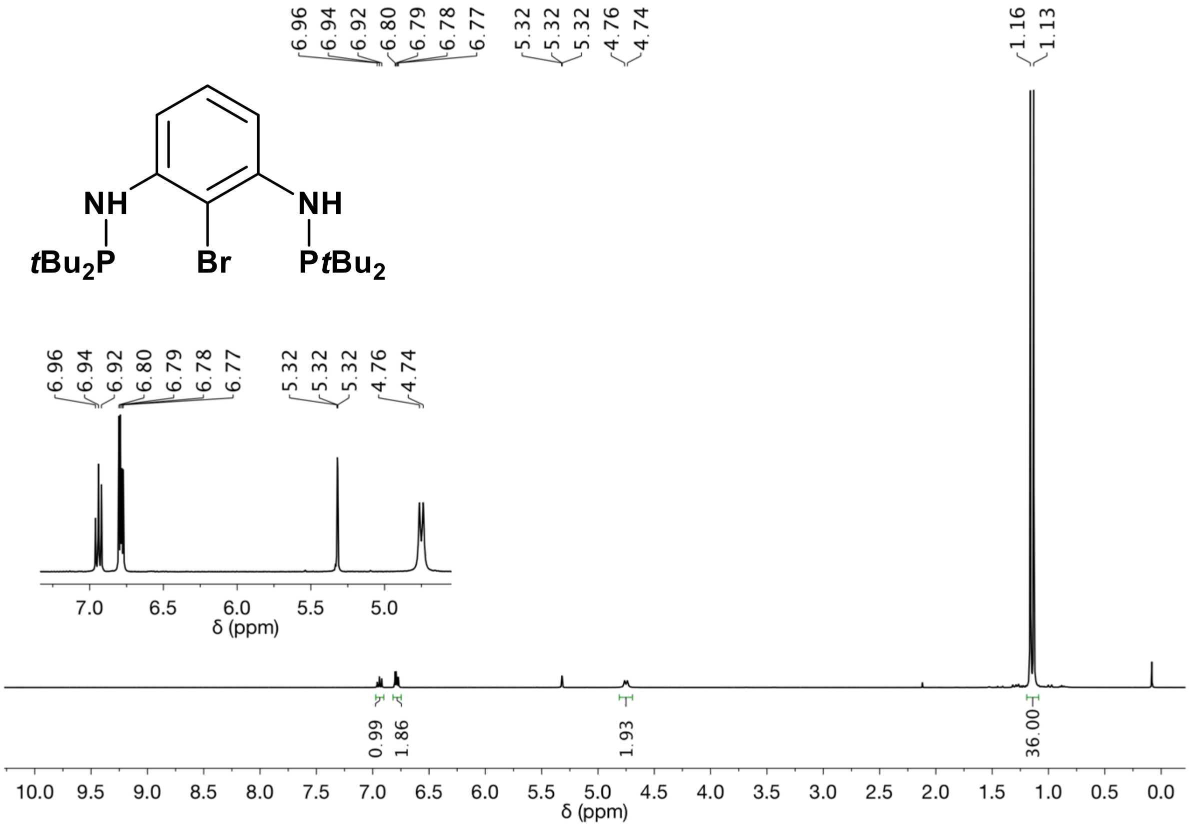

$^{13}C\{^{1}H\}$-NMR (101 MHz, CD_2Cl_2, 20 °C): PCP^{NH}-*t*Bu-Br (**1d**)

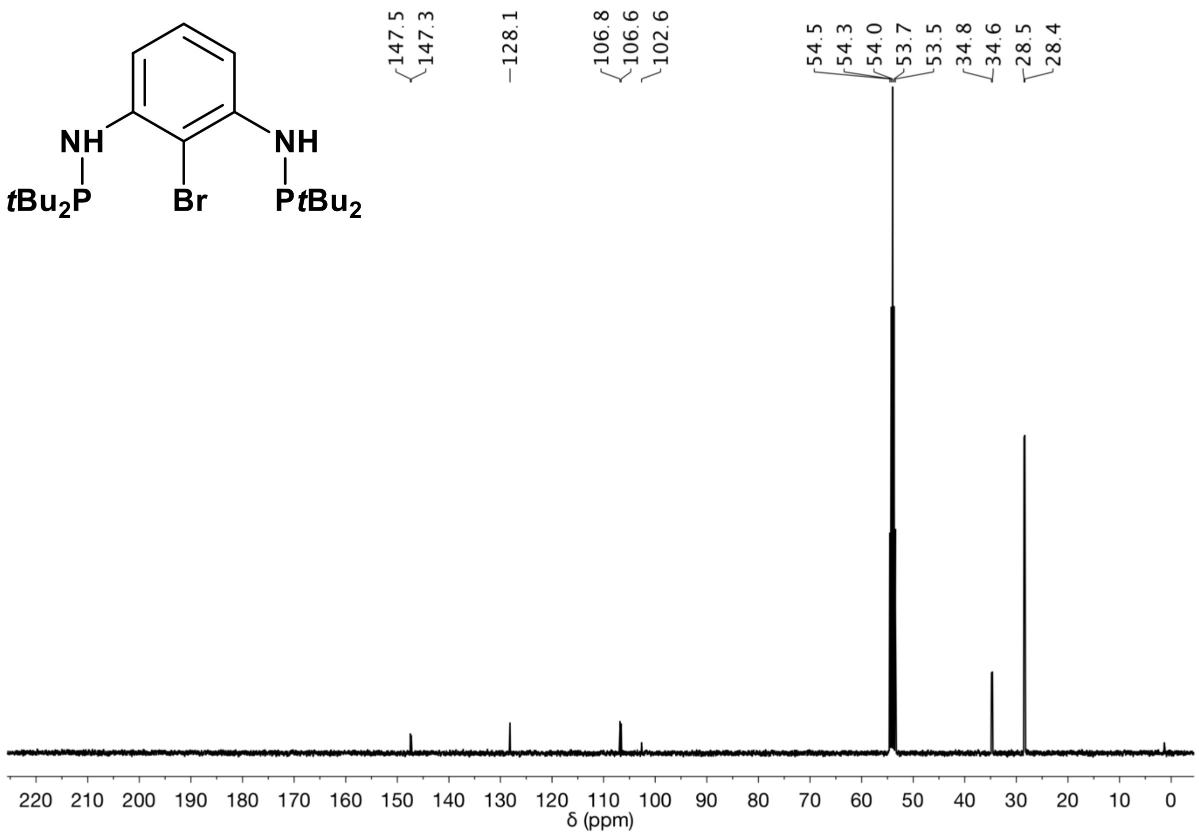

$^{31}P\{^{1}H\}$-NMR (162 MHz, CD_2Cl_2, 20 °C): PCP^{NH}-*t*Bu-Br (**1d**)

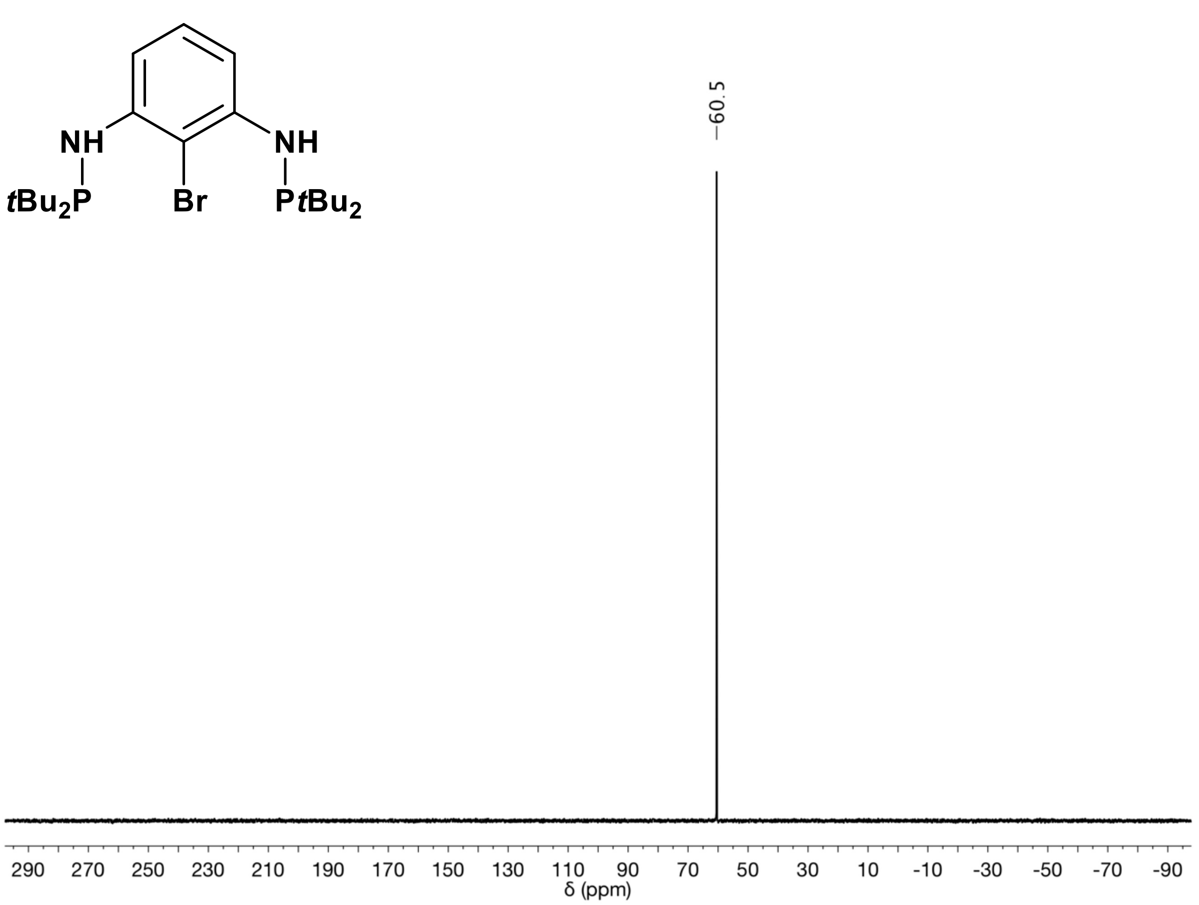

^{1}H-NMR (400 MHz, CD_2Cl_2, 20 °C): [Co^I(PCP^{NH}-*i*Pr)$(CO)_2$] (**3**)

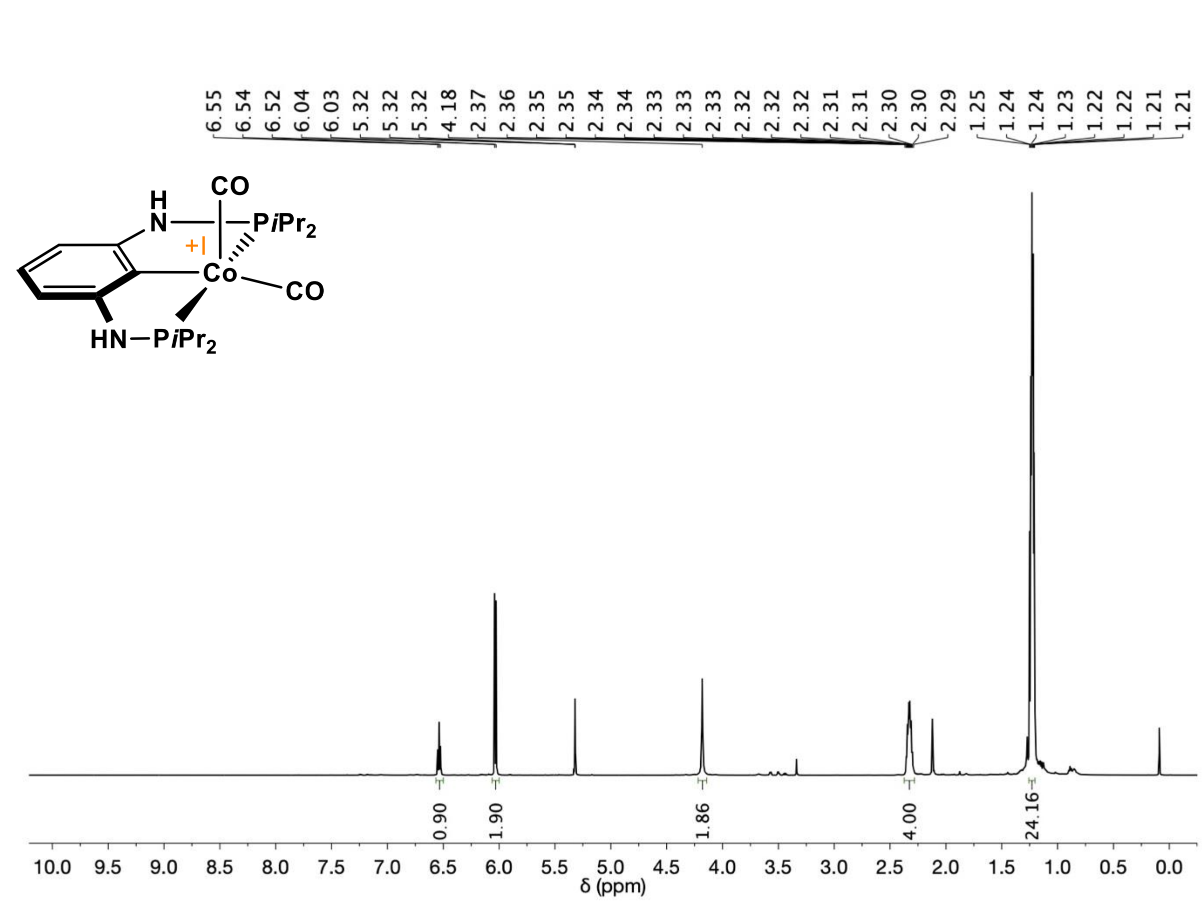

^{13}C{^{1}H}-NMR (101 MHz, CD_2Cl_2, 20 °C): [Co^I(PCP^{NH}-*i*Pr)$(CO)_2$] (**3**)

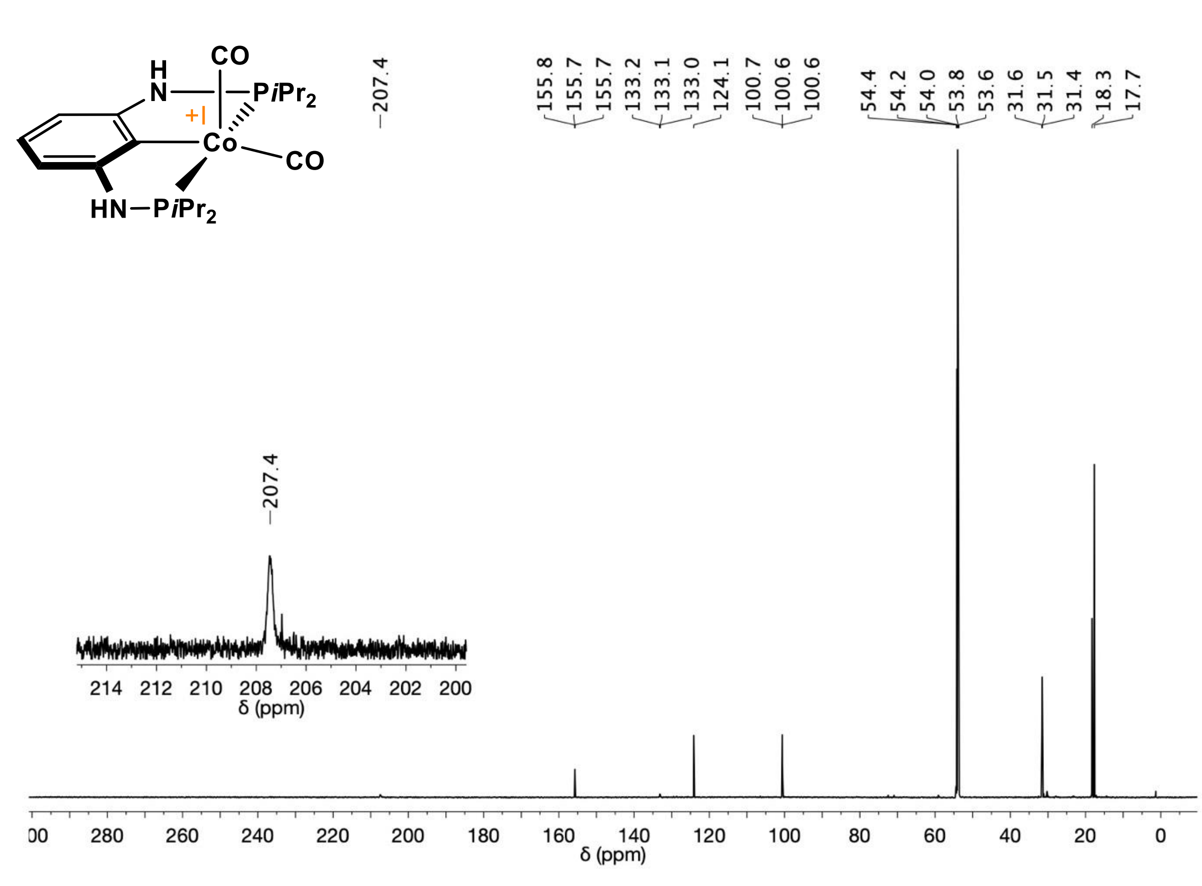

$^{31}P\{^{1}H\}$-NMR (162 MHz, CD_2Cl_2, 20 °C): [Co^{I}(PCP^{NH}-*i*Pr)$(CO)_2$] (**3**)

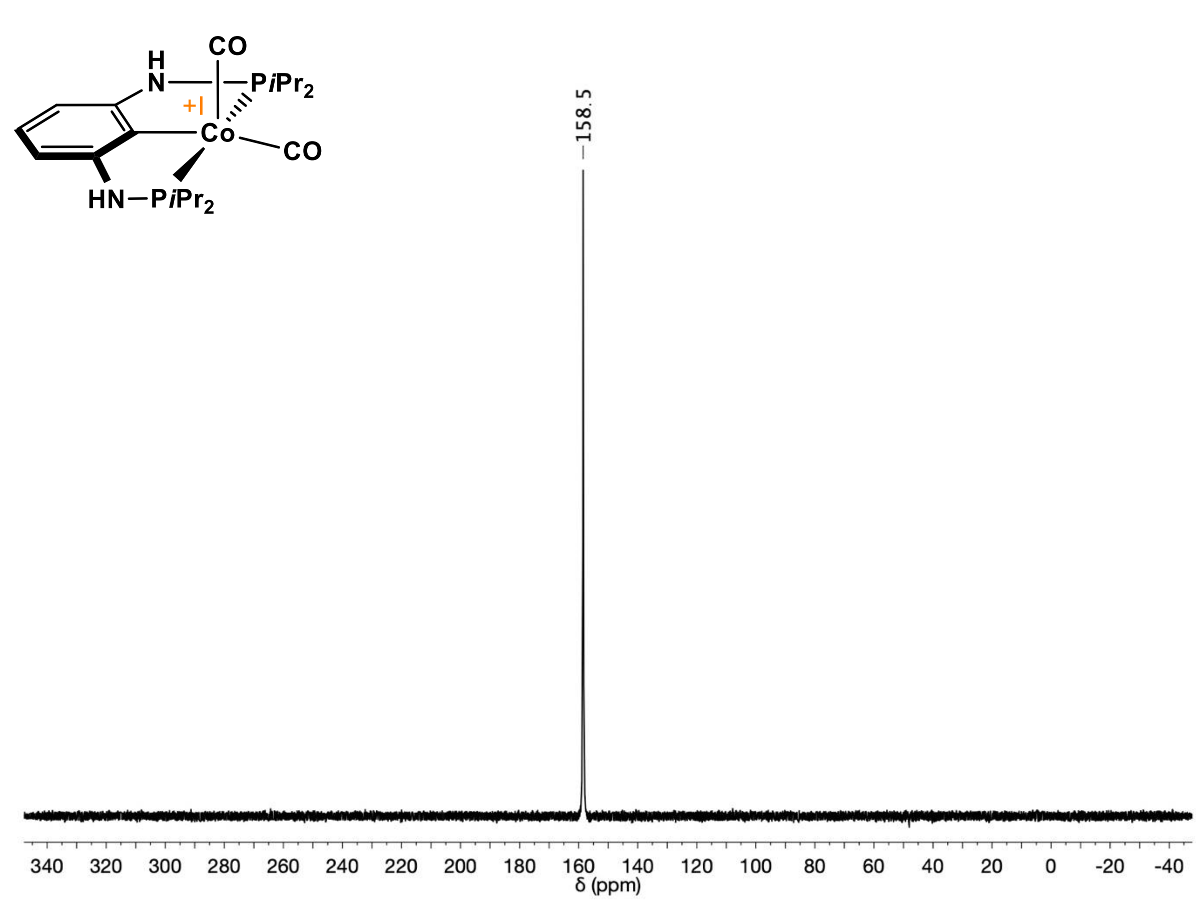

$^{31}P\{^{1}H\}$-NMR (162 MHz, CD_2Cl_2, 20 °C): [Co^{I}(PCP^{NH}-*t*Bu)$(CO)_2$] (**4**)

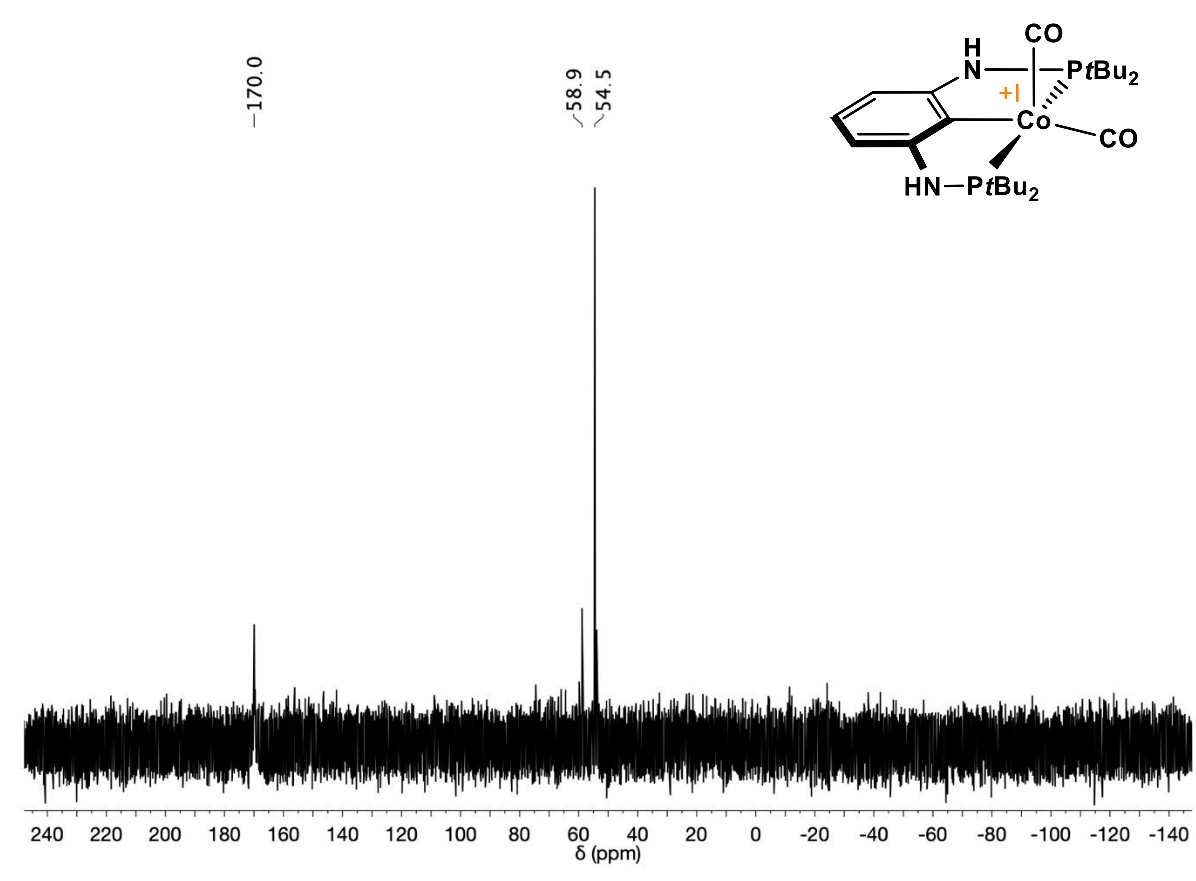

^{1}H-NMR (600 MHz, C_6D_6, 20 °C): [Co^{I}(PCP^{CH_2}-*i*Pr)(CO)$_2$] (**8**)

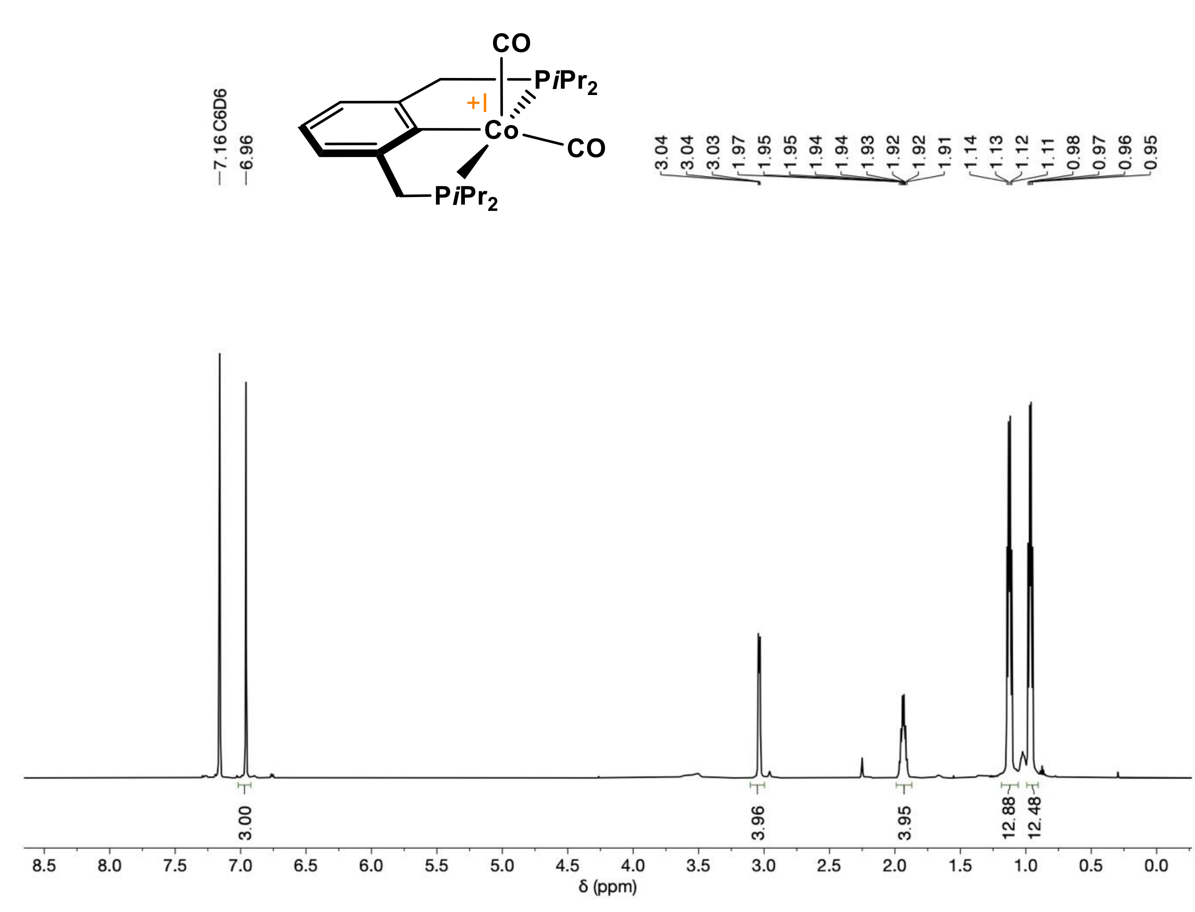

^{13}C{^{1}H}-NMR (151 MHz, CD_2Cl_2, 20 °C): [Co^{I}(PCP^{CH_2}-*i*Pr)(CO)$_2$] (**8**)

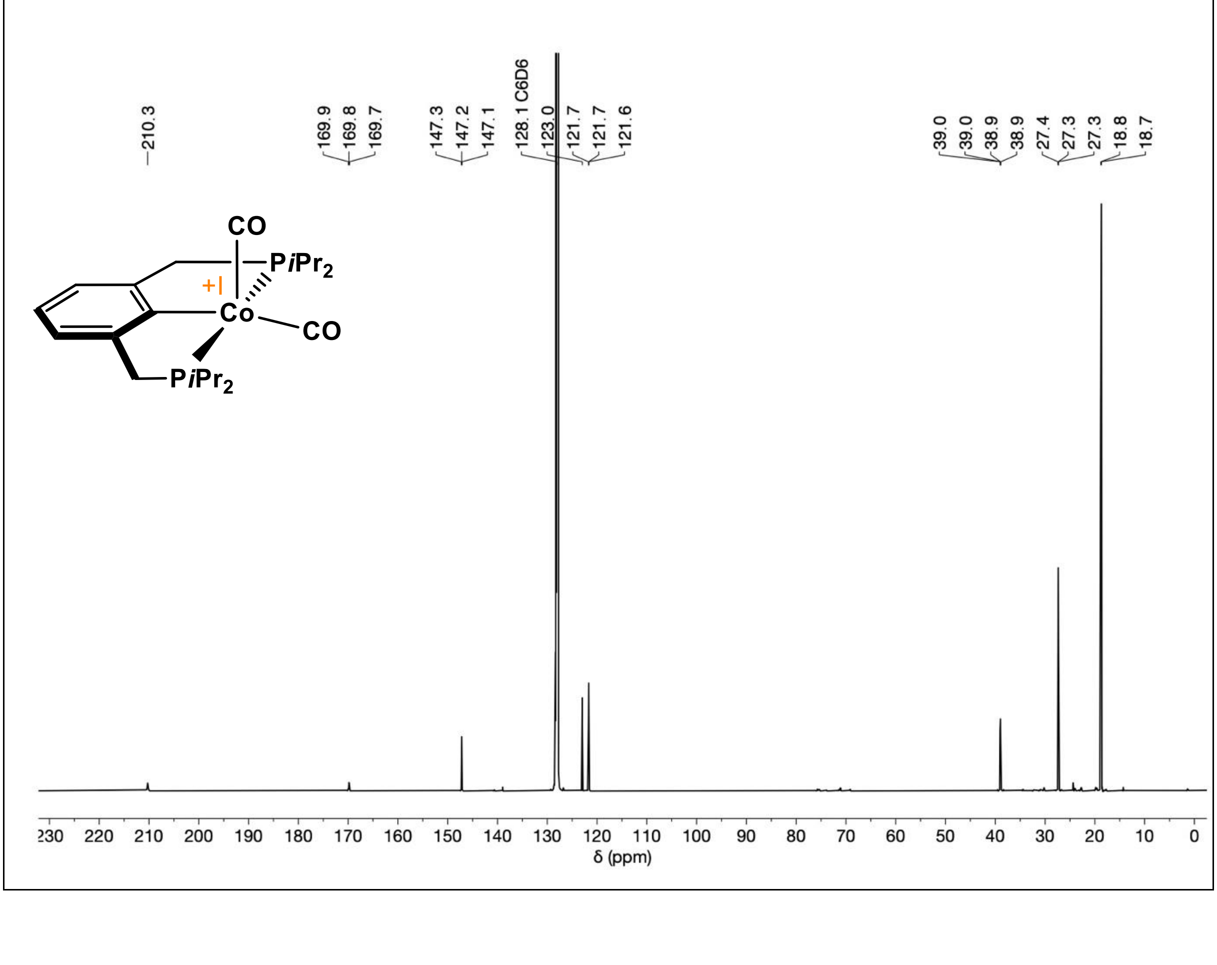

$^{31}P\{^{1}H\}$-NMR (243 MHz, CD_2Cl_2, 20 °C): $[Co^{I}(PCP^{CH_2}\text{-}i\mathrm{Pr})(CO)_2]$ (**8**)

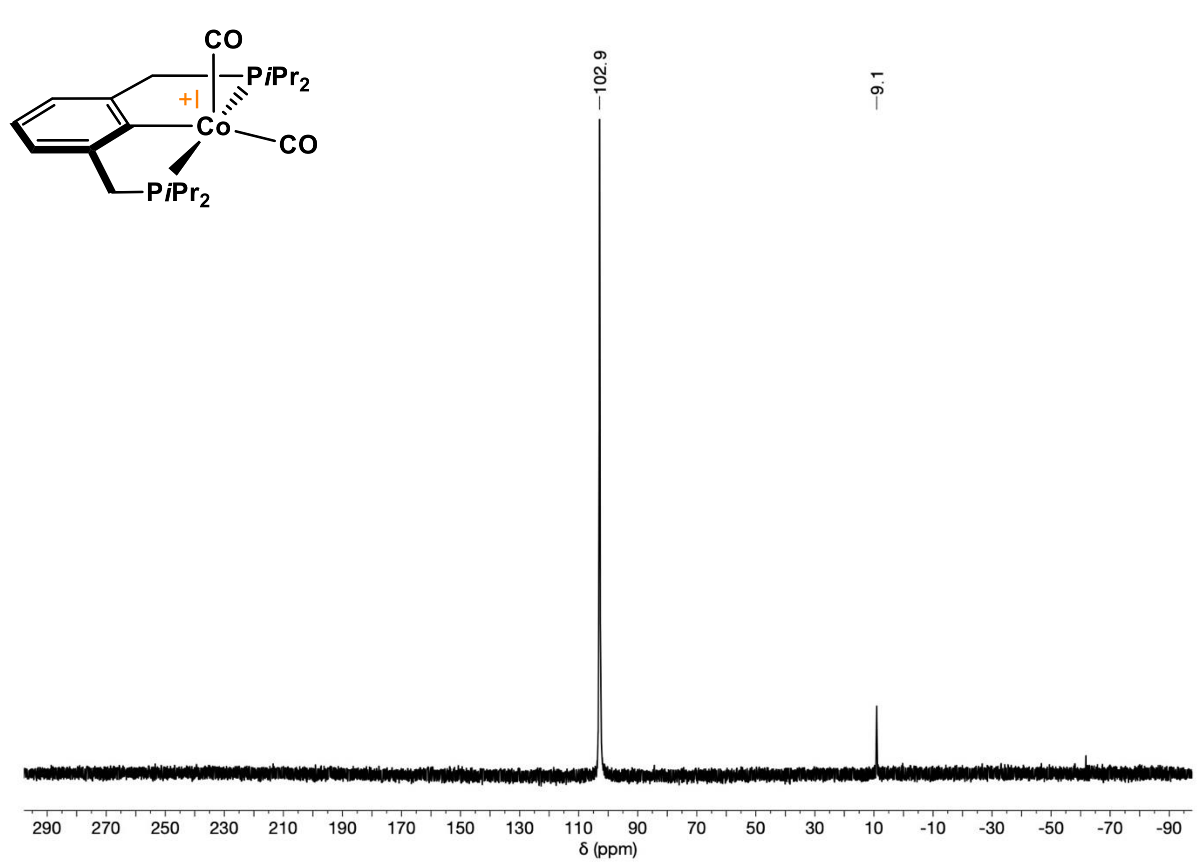

^{1}H-NMR (400 MHz, C_6D_6, 20 °C): $[Co^{I}(PCP^{CH_2}\text{-}t\mathrm{Bu})(CO)_2]$ (**9**)

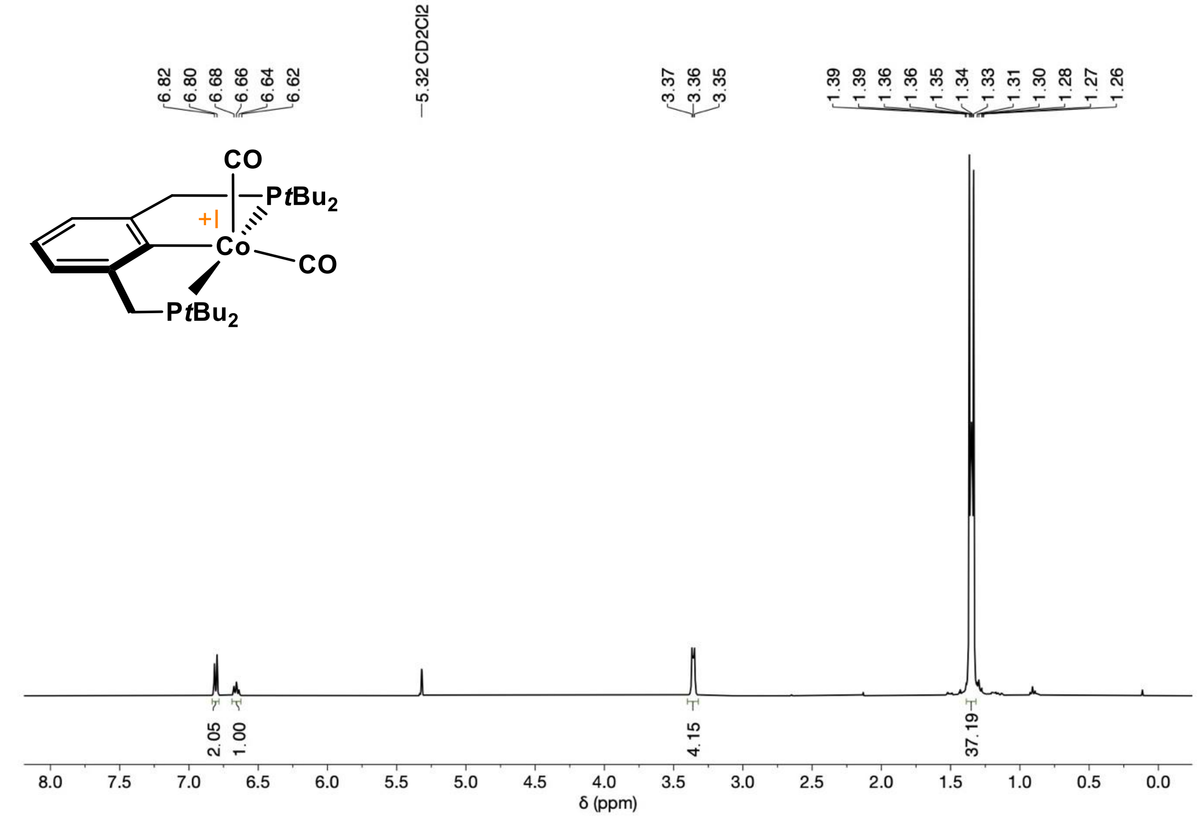

$^{13}C\{^{1}H\}$-NMR (101 MHz, CD_2Cl_2, 20 °C): [Co^{I}(PCP^{CH_2}-*t*Bu)$(CO)_2$] (**9**)

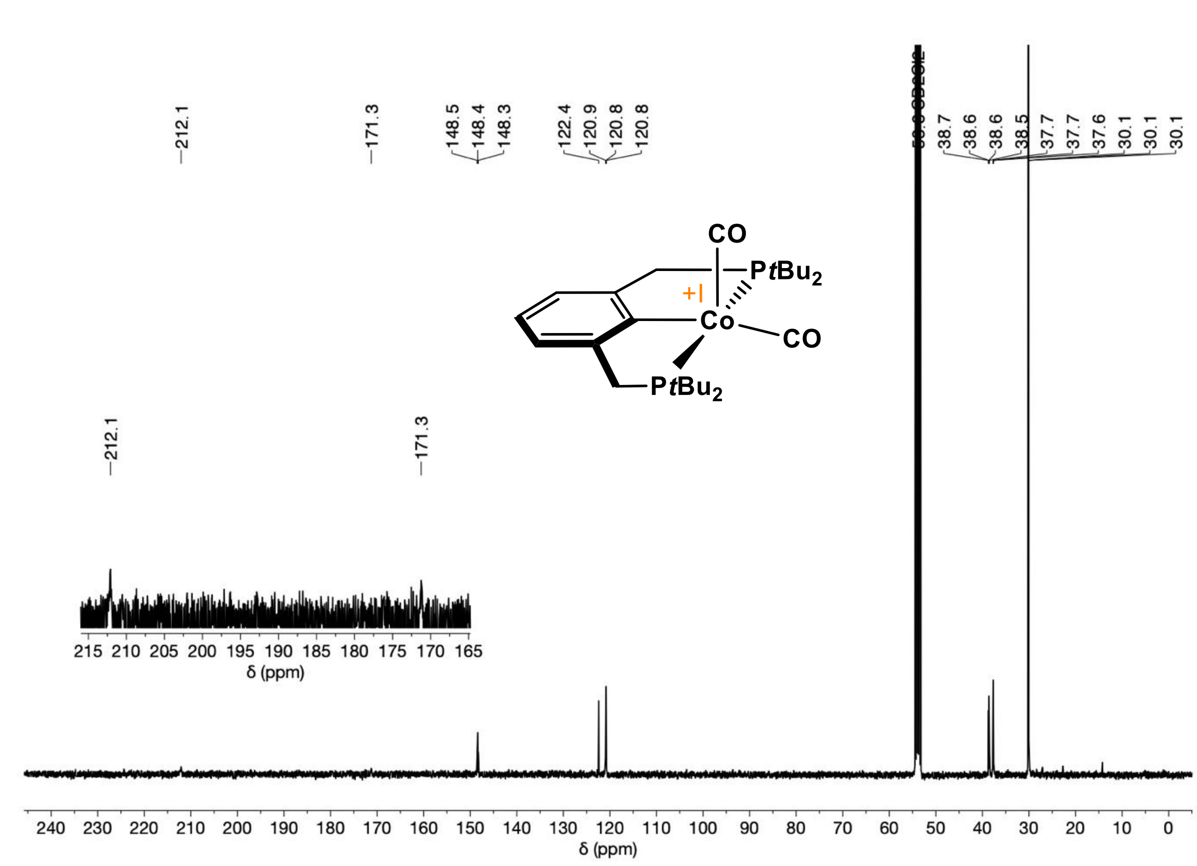

$^{31}P\{^{1}H\}$-NMR (162 MHz, CD_2Cl_2, 20 °C): [Co^{I}(PCP^{CH_2}-*t*Bu)$(CO)_2$] (**9**)

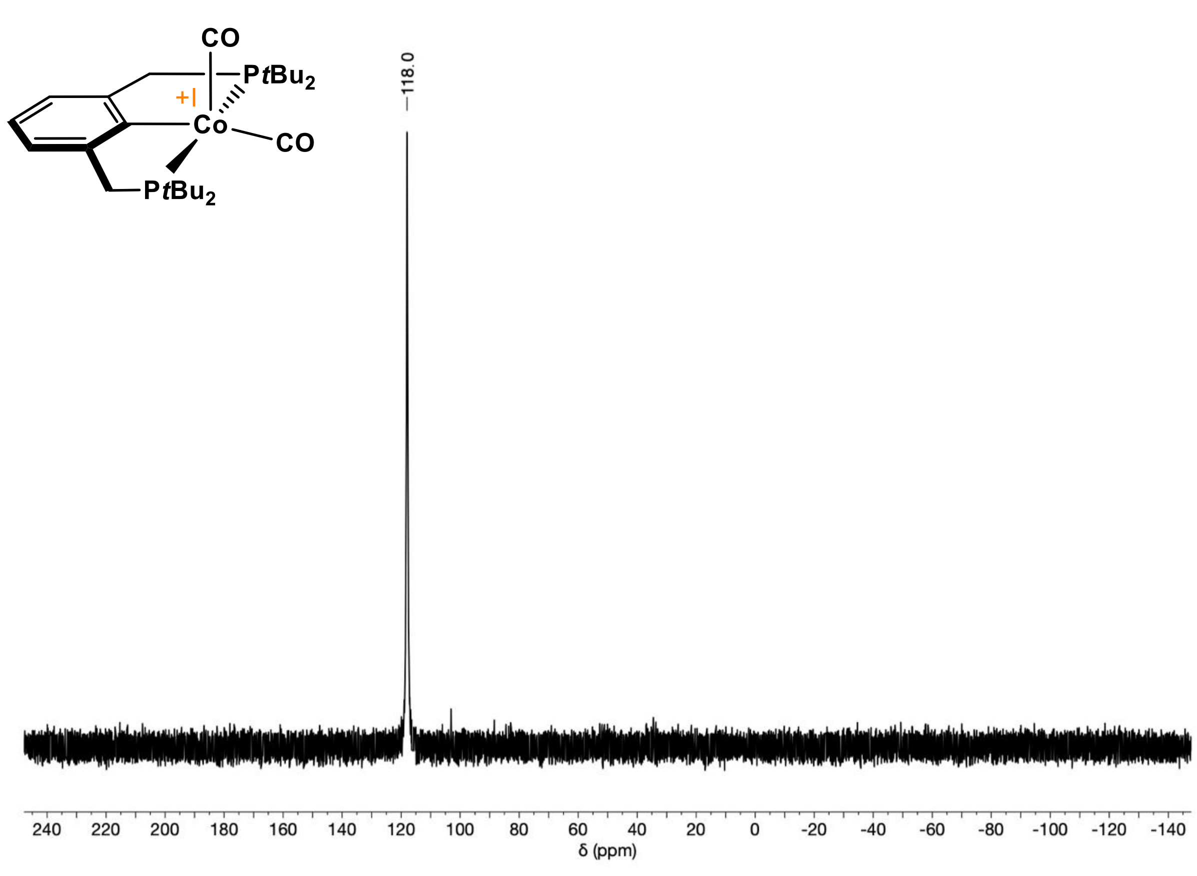

Supplement: Supplementary file 2 — Supplementary file2 (PDF 1501 kb) [file 706_2023_3123_MOESM2_ESM.pdf]
